# Genome-wide structural modelling of TCR-pMHC interactions

**DOI:** 10.1186/1471-2164-14-S5-S5

**Published:** 2013-10-16

**Authors:** I-Hsin Liu, Yu-Shu Lo, Jinn-Moon Yang

**Affiliations:** 1 Institute of Bioinformatics and Systems Biology, National Chiao Tung University, Hsinchu, 30050, Taiwan; 2 Department of Biological Science and Technology, National Chiao Tung University, Hsinchu, 30050, Taiwan

## Abstract

**Background:**

The adaptive immune response is antigen-specific and triggered by pathogen recognition through T cells. Although the interactions and mechanisms of TCR-peptide-MHC (TCR-pMHC) have been studied over three decades, the biological basis for these processes remains controversial. As an increasing number of high-throughput binding epitopes and available TCR-pMHC complex structures, a fast genome-wide structural modelling of TCR-pMHC interactions is an emergent task for understanding immune interactions and developing peptide vaccines.

**Results:**

We first constructed the PPI matrices and *i*Matrix, using 621 non-redundant PPI interfaces and 398 non-redundant antigen-antibody interfaces, respectively, for modelling the MHC-peptide and TCR-peptide interfaces, respectively. The *i*Matrix consists of four knowledge-based scoring matrices to evaluate the hydrogen bonds and van der Waals forces between sidechains or backbones, respectively. The predicted energies of *i*Matrix are high correlated (Pearson's correlation coefficient is 0.6) to 70 experimental free energies on antigen-antibody interfaces. To further investigate *i*Matrix and PPI matrices, we inferred the 701,897 potential peptide antigens with significant statistic from 389 pathogen genomes and modelled the TCR-pMHC interactions using available TCR-pMHC complex structures. These identified peptide antigens keep hydrogen-bond energies and consensus interactions and our TCR-pMHC models can provide detailed interacting models and crucial binding regions.

**Conclusions:**

Experimental results demonstrate that our method can achieve high precision for predicting binding affinity and potential peptide antigens. We believe that *i*Matrix and our template-based method can be useful for the binding mechanisms of TCR-pMHC complexes and peptide vaccine designs.

## Background

An adaptive immune response protects an organism from the infection by identifying and killing pathogens [1,2]. It is antigen-specific and allows for a stronger immune response after the recognition of specific "non-self" antigens by the T-cell receptor (TCR) [3]. As an increasing number of high-throughput experiments providing available and reliable binding epitopes related to various TCRs [4-6], a systematic and fast method to search similar complexes (i.e. TCR-pMHC molecules) is an important task for understanding potential immune interactions and developing pathogen vaccines.

Since rapidly increasing three-dimensional structure complexes in Protein Data Bank (PDB), many structure-based works have been proposed to utilize physical interacting interfaces of these complexes to study protein-protein interactions [7-10], MHC-peptide interactions [11,12], and structural systems biology [13-15]. Most of these works [7-9,11,12] used a scoring-based matrix to evaluate the protein-protein and MHC-peptide interface preferences. In addition, sequence-based matrix methods (e.g. SYFPEITHI [16], MAPPP [17], IEDB [18]) have been proposed for predicting peptide-MHC interactions.

Recently, we have proposed a template-based strategy, called PAComplex [19], which is the first method investigating both peptide-MHC and peptide-TCR interfaces to infer peptide antigens and homologous peptide antigens of a query. This study utilized four scoring matrices and one scoring matrix to calculate the binding scores of peptide-MHC (which is similar to protein-protein interface (PPI)) and TCR-peptide (which is similar to antigen-antibody (Ag-Ab) [20,21]) interfaces, respectively. Our previous works showed that four scoring matrices yielded significantly higher accuracies than one scoring matrix for inferring structure-based PPIs [22,23]. The four scoring matrices include sidechain-sidechain and sidechain-backbone van der Waals energies; and sidechain-sidechain and sidechain-backbone hydrogen-bond energies. In addition, two main factors that deteriorate the performance of PAComplex using one-matrix scores are (i) the hydrogen-bond energies and van der Waals interactions were considered as the same and (ii) the sidechain-sidechain and sidechain-backbone interactions were not discriminated. For example, we observed that the average experimental energies of the residues forming hydrogen bonds and van der Waals interactions 2.54 and 1.08, respectively, based on 70 mutated residues on Ag-Ab interfaces.

To address these issues, we proposed four-matrices scoring function to enhance one-matrix scoring function to infer the peptide antigens using TCR-pMHC complex structures. The major enhancements are as follows: 1) four scoring matrices (named *i*Matrix) can predict template-based binding energies of TCR to pMHC interfaces by separating the van der Waals (vdW) forces from special bonding forces; 2) *i*Matrix discriminates sidechain-sidechain and sidechain-backbone interactions into two matrices; 3) a fast and genomic-scale searching method for identifying peptide antigens of a template TCR-pMHC structure; 4) *i*Matrix highlights the critical hydrogen bonds for key interacting residues between TCR-pMHC compexes.

To validate the reliability and enlarge the number of potential antigens, we evaluate our methods on experimental free energy data and 389 complete pathogen genomes. Experimental results indicated that *i*Matrix can achieve a high correlation of the binding interface energies. In addition, the homologous peptide antigens derived from *i*Matrix have a high precision value and keep the hydrogen bonds based on template then they should be the reliable peptide antigens. The *i*Matrix also reveals detailed interacting models for TCR-pMHC complexes distinctively and display the mechanisms of crucial binding regions. Furthermore, the *i*Matrix scoring function can provide important insights into heightened immunogenicity derived from the potential peptide antigens or epitopes and can infer valuable vaccine design for clinical trials.

## Methods

### Overview for genome-wide structural modelling of TCR-pMHC interactions

According to our previous study, the homologous peptide antigen (p') of the peptide (p) in template complex as follows: (1) p and p' can be bound by the same MHC forming pMHC and p'MHC, respectively, with the significant interface similarity (*Z_MHC _*≥ 1.645); (2) pMHC and p'MHC can be recognized by the same TCR with significant peptide-TCR interface similarity (*Z_TCR _*≥ 1.645); and (3) TCR-pMHC and TCR-p'MHC share significant complex similarity (joint *Z*-value ≥ 4.0). The joint *Z*-value (*J_z_*) is defined as

(1) $$J_{z}=\sqrt{Z_{MHC}\times Z_{TCR}}$$

Here, *J_z _*≥ 4.0 is considered a significant similarity according to the statistical analysis of 17 TCR-pMHC structure complexes (i.e. TCR-peptide-HLA-A0201 complexes); 80,057 experimental peptide antigens; and ≥ 10^8 ^peptide candidates derived from 864,628 protein sequences in 389 pathogens.

**Figure 1:**
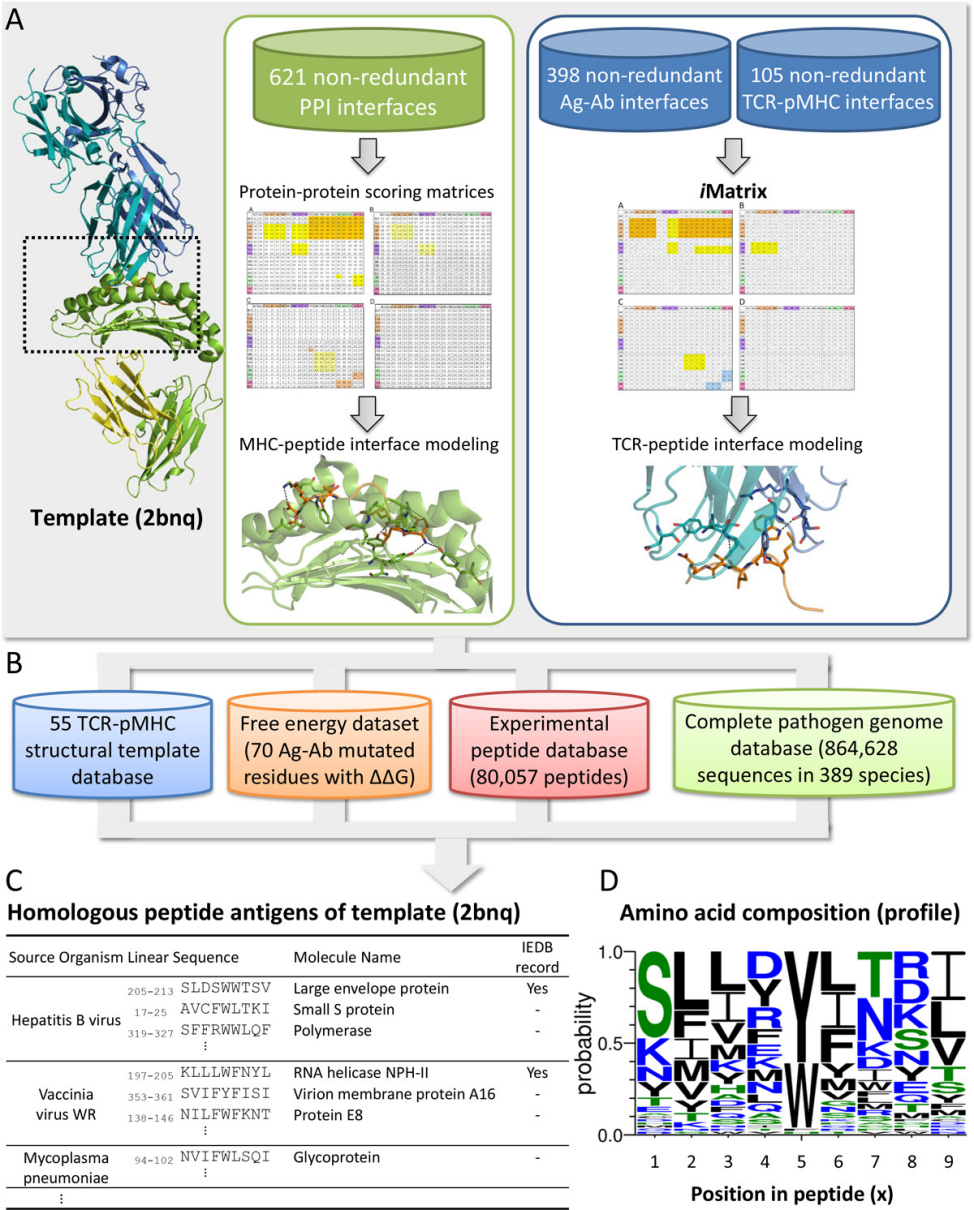
**Overview of the iMatrix and homologous peptide antigens**. (A) Data sets for constructing PPI matrices and *i*Matrix. The MHC-peptide and TCR-peptide interfaces are modelling by PPI matrices and *i*Matrix, respectively. (B) The template-based scoring functions infer the homologous peptide antigens through structural templates, experimental peptides, and complete pathogen genome databases. (C) Homologous peptide antigens of the template (e.g., PDB entry: 2bnq) by searching the experimental peptides and complete pathogen genome databases. (D) Amino acid profiles of the homologous peptide antigens of the template (2bnq).

Figure 1 shows the main procedures of genome-wide structural modelling of TCR-pMHC interactions using the *i*Matrix and PPI matrices. According to a general mathematical structure constructing a standard log-odds matrix [24-26], we first constructed the PPI matrices from the 621 non-redundant PPI interfaces, and *i*Matrix from 398 non-redundant Ag-Ab interfaces and 105 non-redundant TCR-pMHC interfaces. The matrices of PPI and *i*Matrix are used for modelling the MHC-peptide and TCR-peptide interfaces, respectively (Figure 1A). We then utilized 70 point mutations in four Ag-Ab interfaces recorded in the Alanine Scanning Energetics database (ASEdb) [27] to evaluate the relationship between *i*Matrix and experimental free energies (Figure 1B). To further investigate the reliabilities of homologous peptide antigens derived from the template-based scoring function, we prepared 55 TCR-pMHC complexes as templates. We inferred the homologous peptide antigens of each TCR-pMHC complex (e.g. PDB entry 2bnq [28]) from an Immune Epitope Database (IEDB) (80,057 peptides in 2,287 species) and a complete pathogen genome database (≥ 10^8 ^peptide antigen candidates with *J_z _*≥ 1.645 derived from 864,628 protein sequences of 389 pathogens) (Figure 1B and 1C). Here, these 389 pathogens (e.g., bacteria, archaea, and virus) recorded in both IEDB [6] and UniProt [29] databases and their respective complete genomes collected from UniProt database. For each peptide antigen family, we measure the amino acid composition and conservation at each position (Figure 1D) by WebLogo program [30]. Finally, *i*Matix could provide the peptide antigens from a large-scale pathogen database, the TCR-pMHC interaction models, and the peptide antigen families with conserved amino acids.

### Scoring function and iMatrix

**Figure 2:**
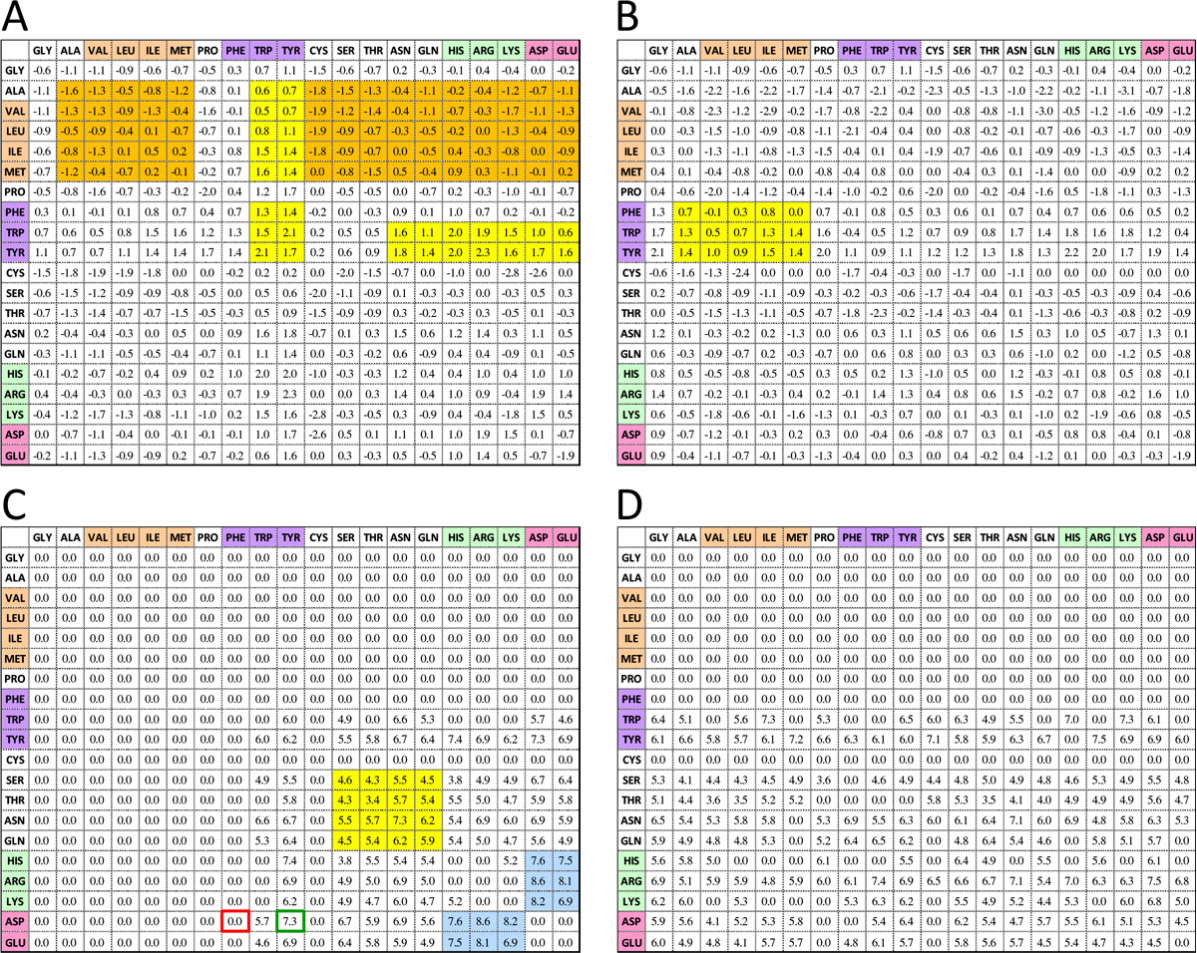
**Four knowledge-based scoring matrices of iMatrix**. (A) Sidechain to sidechain van der Waals scoring matrix; (B) Sidechain to backbone van-der Waals scoring matrix; (C) Sidechain to sidechain special-bond scoring matrix; (D) Sidechain to backbone special-bond scoring matrix. The sidechain to sidechain scoring matrices are symmetric. For sidechain to backbone matrices, y-axis denotes side chain and x-axis denotes backbone. We discard backbone-backbone matrixes because the backbone-backbone interacting forces are constant in our template-based method.

We have recently proposed a template-based scoring function to determine the protein-protein interactions (PPIs) derived from a 3D-dimer structure [22,23]. For the peptide-MHC and peptide-TCR interaction, the scoring function is defined as

(2) $$E_{Total}=E_{vdW}+E_{SF}+E_{sim}$$

where *E_vdW _*is the van der Waal's energy; *E_SF _*is the special energy (i.e. hydrogen-bond energy and electrostatic energy); and *E_sim _*refers to the peptide similarity score between query and template. In PAcomplex, The *E_vdW _*and *E_SF _*of peptide-TCR interfaces are calculated by the one-matrix (Fig. S1 in Additional file 1). However, the *E_vdW _*and *E_SF _*of peptide-MHC and peptide-TCR interfaces are calculated by the four matrices of PPI and *i*Matrix, respectively, in this study. The *E_vdW _*and *E_SF _*are given as

(3) $$E_{vdW}=\overset{CP}{\underset{i,j}{\sum }}\left(Vss_{ij}+Vsb_{ij}+Vsb_{ji}\right)$$

(4) $$E_{SF}=\overset{CP}{\underset{i,j}{\sum }}\left(SFss_{ij}+SFsb_{ij}+SFsb_{ji}\right)$$

where *CP *denotes the number of the aligned-contact residues of query peptide and the hit template peptide. *Vss_ij _*and *Vsb_ij _*(*Vsb_ji_*) are the sidechain to sidechain and sidechain to backbone vdW energies between residues *i *(in peptide side) and *j *(in TCR or MHC side), respectively. *SFss_ij _*and *SFsb_ij _*(*SFsb_ji_*) are the sidechain to sidechain and sidechain to backbone special interacting energies between residue *i *(in peptide side) and *j *(in TCR or MHC side), respectively, if the contact-pair residues *i *and *j *form the special bonds (i.e. hydrogen bond, salt bridge, or electrostatic energy) in the template structure. The vdW energies (*Vss_ij_*, *Vsb_ij_*, and *Vsb_ji_*) and special interacting energies (*Tss_ij_*, *Tsb_ij_*, and *Tsb_ji_*) of peptide-MHC and peptide-TCR can be obtained from PPI matrices (Fig. S2 in Additional file 2) and *i*Matrix (Figure 2), including sidechain-sidechain (Figs. S2A and 2A) and sidechain-backbone van der Waals scoring matrices (Figs. S2B and 2B in Additional file 2); and sidechain-sidechain (Figs. S2C and 2C in Additional file 2) and sidechain-backbone special-bond scoring matrices (Figs. S2D and 2D in Additional file 2). The sidechain-sidechain scoring matrices are symmetric and sidechain-backbone scoring matrices are non-symmetric.

Following calculation of the interaction scores (*E_tot_*), these scores are transformed into *Z*-values (i.e., *Z_MHC _*and *Z_TCR_*) of peptide-MHC and peptide-TCR interfaces using the mean and standard deviation derived from 10,000 random interfaces by mutating each peptide position. For a TCR-pMHC template collected from the Protein Data Bank (PDB) [31], these 10,000 random interfaces are generated by substituting with another amino acid according to the amino acid composition derived from UniProt [29]. Finally, we computed *J_Z _*(Equation 1) of the TCR-pMHC complex.

### Data set of constructing iMatrix

**Figure 3:**
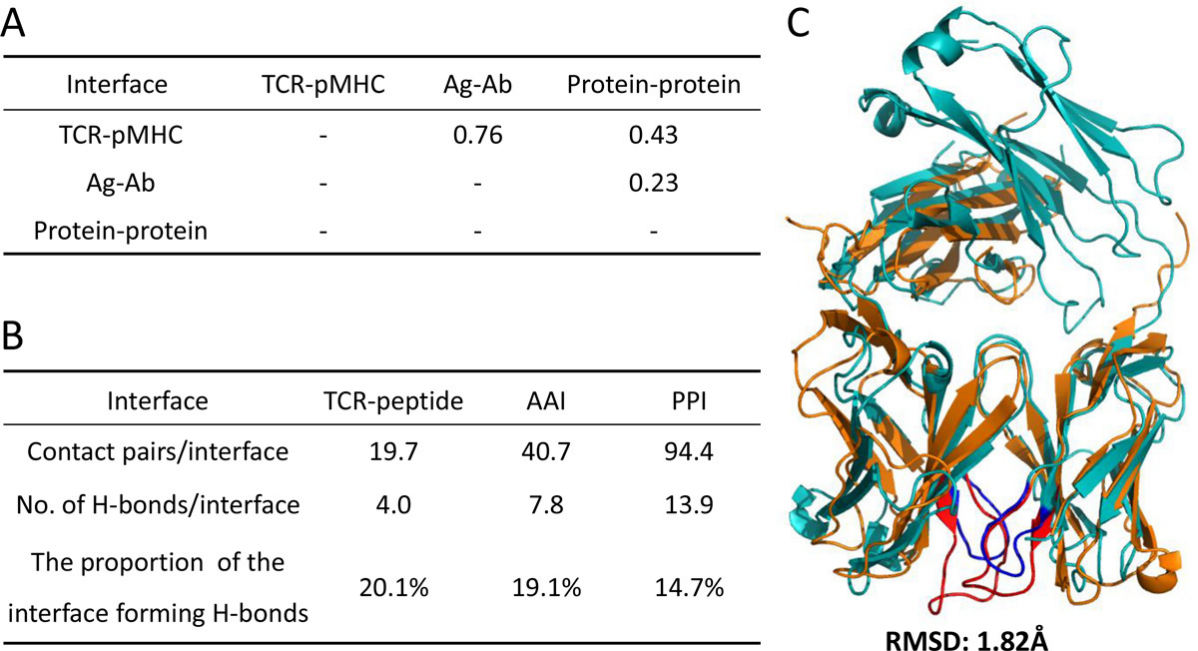
**Comparison between the TCR-pMHC and antigen-antibody interfaces**. (A) Pearson's correlation coefficient of 20 amino acid preferences within paired interfaces among TCR-peptide, antigen-antibody, and protein-protein interfaces. (B) Hydrogen bonding proportions in contact pairs for three kinds of interfaces. (C) Structure alignment of TCR-pMHC (PDB entry: 1ao7) and antigen-antibody (PDB entry: 1jps) complexes using MultiProt. αβTCR chains (orange) are aligned to heavy and light chains of antibody (light blue) and the RMSD is 1.82 Å.

Because of the different properties between protein-protein and TCR-pMHC interfaces, the scoring matrices for describing PPIs [23] are unsuitable for modelling TCR-pMHC. For modelling TCR-pMHC interactions, we collected a great quantity of co-crystal structures of TCR-pMHC complexes which were only 55 MHC class I and 9 MHC class II in PDB (January 2012). In addition, these sequences and structures are often very similar. Conversely, the number and sequences of co-crystal antigen-antibody (Ag-Ab) structures are significantly large and diverse, respectively. According to the comparison between Ag-Ab and TCR-pMHC interfaces (Figure 3), the TCRs and Fab fragments of antibodies often share similar structures on the binding sites (e.g. complementarity determining regions (CDRs)) [32].

Therefore, we built a dataset, consists of 398 Ag-Ab interactions, to generate the *i*Matrix for modelling TCR-pMHC interfaces (Figure 1A and 2). We first manually collected 679 crystal structures of Ag-Ab complexes from the PDB (April 2012) at a resolution less than or equal to 3Å. The binding interfaces consist of one protein antigen and one antibody whose fragments outside of variable regions are excluded from the analysis. All protein chains were pairwise aligned to make non-redundant sequence set using BLASTClust [33]. Finally, the 229 Ag-Ab complexes (Table S1 in Additional file 3) with 398 Ag-Ab interfaces (Table S2 in Additional file 4) were collected in this set.

### Experimental free energy dataset

To further investigate the relationship between the predicted energy and experimental free energy, we collected 70 mutated residues, which are contact residues in Ag-Ab interfaces in 4 structural complexes from the ASEdb (Table S3 in Additional file 5). The Alanine Scanning Energetics database is a repository for energetics of sidechain interactions determined by alanine-scanning mutagenesis [27]. ASEdb gives the corresponding ΔΔG value representing the change in free energy of binding upon mutation to alanine for each experimentally mutated residue.

### The experimental peptide antigens derived from IEDB

To further evaluate the reliability of homologous peptide antigen derived from the *i*Matrix, we collected the 80,057 experimental peptides from the IEDB (January 2013) for 389 pathogens; and 17 TCR-pMHC complexes (i.e. TCR-peptide-HLA-A0201, Table S4 in Additional file 6) from the PDB. Then, we filtered 4,987 positive nonamers and 4,322 negative nonamers of TCR-peptide-HLA-A0201. Here, the definition of positive records is at least one positive measurement in T cell response or MHC binding assays; negative records are data with only negative measurements. We also prepared the H-2-Kb (*Mus musculus*) and H-2-Ld (*Mus musculus*) alleles for validation of *i*Matrix.

In addition, in these 389 pathogens, the *vaccinia virus *has the largest amount (19.7%) of experimental records in the IEDB, including 1,131 positive nonamers and 706 negative nonamers. Here, the complete genomes of *vaccinia virus *are 320 proteins recorded in UniProt [29], and we processed them into 79,157 nonamers (56,030 non-redundant nonamers). This *vaccinia virus *subset was used in case studies.

## Results and discussion

### *i*Matrix

The high scores in four scoring matrices of *i*Matrix are often superior frequency of interacting residue pairs. The sidechain-sidechain scoring matrices are symmetric. In sidechain-backbone matrices (e.g., Figure 2B, 2D, S2B, and S2D in Additional file 2), y-axis denotes side chain and x-axis denotes backbone. The interacting score is set to zero if the frequency of an entry (a contacted pair residue) is 0.

For vdW scoring matrices of *i*Matrix (Figure 2A and 2B), the scores are high when aromatic residues (i.e., Phe, Trp, and Tyr) interact to aromatic and large-sidechain residues (e.g., Met, Ile, and Arg). The result is consistent to the previous results that residues Tyr and Trp play key roles in epitopes and paratopes [34]. Conversely, the result is different from the vdW matrices of protein-protein interactions [23], which the aromatic residues only prefer interacting aromatic residues (yellow blocks; Figs. S2A and S2B in Additional file 2). Additionally, the scores are low while aliphatic residues (i.e. Ala, Val, Leu, Ile, Met, and Pro) interact to the other residues (orange blocks; Figure 2A) for immune complexes. The results are significantly different from the vdW matrices of protein-protein interfaces (yellow blocks; Figure S2A in Additional file 2).

For special-bond scoring matrices (Figure 2C and 2D), the scores (blue blocks in Figure 2C) are significantly high when the residues with polar groups (i.e. Tyr, Trp, Asn, and Gln; yellow blocks) or basic residues (i.e. His, Arg, and Lys) interact to acidic residues (i.e. Asp and Glu). These results are consistent to the results of protein-protein interfaces (orange block; Figure S2C in Additional file 2).

### TCR-pMHC interfaces

Based on our previous researches, the template-based scoring function achieves good agreement for the binding affinity in PPIs [13]. The novel knowledge-based matrices were derived using a general mathematical structure [24] from a non-redundant set of 621 3D-dimer complexes proposed by Glaser *et al. *[35]. This dataset is composed of 217 heterodimers and 404 homodimers and the sequence identity is less than 30% to each other. However, the matrices may not be applied to model TCR-peptide binding because previous studies have indicated that the TCR-pMHC interface resembles Ag-Ab interactions [20,21]. We compared the TCR-pMHC, Ag-Ab, and protein-protein interfaces and presented our observations in global and local views. The TCR-pMHC and Ag-Ab co-crystal complexes were collected from the PDB (April 2012), including 105 and 398 non-redundant interfaces, respectively. PPIs set derived from 621 non-redundant interfaces [23,35].

### Amino acid preferences

To display an overall measure of the interaction frequencies of each amino acid with all the residues of the complementary interface, we calculated the preferences of amino acids in three kinds of interfaces, including TCR-pMHC, Ag-Ab, and protein-protein interfaces. The preference (*P_i_*) of the amino acid type *i *in the molecular interfaces can be calculated by equation (5):

(5) $$P_{i}=\frac{I_{i}}{\sum_{1}^{20}I_{i}}$$

where *I_i _*represnts the numbers of the amino acid type *i *in the interfaces. Next, we derived the interfaces similarity by pairwise comparison using the Pearson's correlation coefficient (PCC). The PCC of 20 amino acid types between any two sets of TCR-pMHC, Ag-Ab, and protein-protein interfaces are shown in Figure 3A. Since the strong positive PCC (0.76) between TCR-pMHC and Ag-Ab interfaces, their amino acid preferences are significantly similar. However, neither TCR-pMHC nor Ag-Ab interfaces are similar to protein-protein interfaces. This result indicates that the composition of TCR-pMHC and Ag-Ab interfaces seems to resemble each other closely.

### Propensities of interface sizes and hydrogen bonds

We then gathered the sizes and proportions of hydrogen bonds (H-bonds) among TCR-pMHC, Ag-Ab, and protein-protein interfaces to analyse their properties. The average numbers of interacting residue pairs of TCR-pMHC (19.7 contact pairs/interface) and Ag-Ab (40.7 contact pairs/interface) interfaces are significantly less than the one of the protein-protein interfaces (94.4 contact pairs/interface) (Figure 3B). This informs that such immune-related binding regions are small than average. Interestingly, the H-bonds proportions of TCR-pMHC interfaces (20.1%) and Ag-Ab interfaces (19.1%) are slight higher than protein-protein interfaces (14.7%). H-bonds are extremely important in biological systems and play a key role in the structure of polymers, both synthetic and natural. These results suggest that although the TCR-pMHC and Ag-Ab interfaces are short and discontinuous, H-bonds might contribute a crucial part.

### Local structural alignment of binding domains

TCR and antibody are composed of six variable loops (CDRs) and have the same domain annotation (i.e. V set domains (antibody variable domain-like)) based on SCOP [36] database. For local analysis the binding regions, we performed a structural alignment of the functional domains in TCR and antibody using MultiProt [37], an efficient and accurate method for local structural pairwise and multiple alignment. Figure 3C shows that the V set domains of TCRs and antibodies share highly structural similarity (in general, RMSD ≤ 2.0 Å). Currently, it is postulated that the CDR3 loops of TCR α and β chains specifically recognize the diversity of bound peptides of pMHC [38] thus play a key role of TCR-pMHC binding. We observed the details of structural alignment and found that CDR3 and contact regions of TCR (Figure 3C, red loops) and antibody (Figure 3C, blue loops) were well aligned together.

### Evaluation of binding affinity

**Figure 4:**
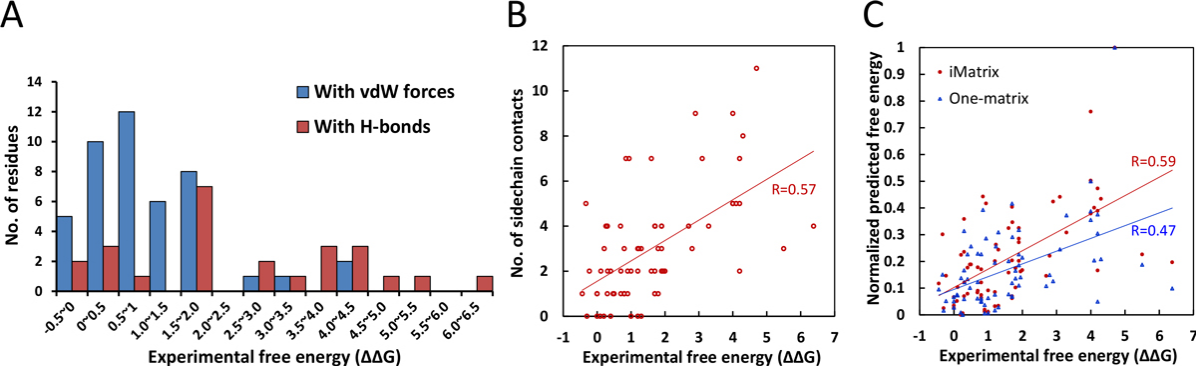
**The evaluation of iMatrix on 70 mutations from the ASEdb**. (A) The distribution of binding energy changes (ΔΔG) based on 70 mutated residues of antigen-antibody interfaces recorded in ASEdb. The mean is 2.54 and standard deviation is 1.84 of the binding free energy for 25 residues forming hydrogen bonds (red bars). Conversely, the mean is 1.08 and standard deviation is 1.03 of 45 residues forming vdW interactions (blue bars). (B) Distribution of free energies for the residues on sidechain interactions. The residues forming more side-chain contacts are often more influenced during the residue mutated into alanine. Pearson correlation coefficient is 0.57 between the ΔΔG and the number of side-chain contact. (C) The Pearson correlation coefficient are 0.59 and 0.47 between 70 experimental free energies (ΔΔG, recorded in ASEdb) and computational scores using iMatrix (red spot) and one-matrix (blue triangle), respectively.

To determine the contribution of a residue to the binding affinity, the alanine-scanning mutagenesis is frequently used as an experimental probe. We selected 70 mutated residues collected from the ASEdb [27] with 4 Ag-Ab complexes whose 3D structures were known. Those mutated residues should position at protein-protein interfaces and be the contact residues. Based on the interacting characteristics, these 70 mutated residues can be divided into two types, including the residues forming hydrogen bonds and the other residues. Among 25 mutated residues forming H-bonds, the ΔΔG values (red bars in Figure 4A; the mean is 2.54 and the standard deviation is 1.84) are significantly higher than 45 mutated residues with vdW interactions (blue bars in Figure 4A; the mean and the standard deviation are 1.08 and 1.03, respectively) and the *p *-value < 0.001. A residue mutation with the ΔΔG > 2.0 is often considered as a hot spot and this residue often contribute extraordinarily high energy [39]. If the side chain of a residue forming H-bonds in the interface, the residue mutated to alanine often breaks this hydrogen bond. For these 70 mutated residues, 48% (12/25) residues forming h-bonds and 9% (4/45) residues with vdW interactions are hot spots due to their ΔΔG > 2.0. Among 4 mutated residues with vdW forces, 3 residues (75%, 2 Phe residues and 1 Trp residue) and their complementary contact residues (2 Tyr residues and 2 Trp residues) form the stack force interactions. This high binding energy is consistent with the high binding scores in vdW scoring matrix (yellow block; Figure 2A). This result implied that the formation of H-bonds in Ag-Ab interfaces indeed dominates the binding energy changes. At the same time, the residues forming more sidechain contacts could from more energy and be more influenced during the residue mutation to alanine which only has a short sidechain. Figure 4B illustrates the relationship between the ΔΔG and the number of sidechain contact. The significant correlation (R = 0.57) implied that the sidechain contact in Ag-Ab interfaces also indeed dominates the binding energy changes.

In addition, *i*Matrix were evaluated on these 70 mutated residues to observe the correlation between experimental ΔΔG values and predicted energies. The PCC between two scoring systems (i.e. *i*Matrix (red) and one matrix used in PAComplex (blue)) and free energies are shown in Figure 4C. The PCC values of *i*Matrix and one matrix are 0.59 and 0.47, respectively. Our results show that the *i*Matrix which separate vdW forces, hydrogen bonds, sidechain contact, and backbone contact could have higher correlation of the binding interface energies. This result is also consistence with the ΔΔG contribution of H-bond and sidechain contact (Figure 4A and 4B). These results imply that *i*Matrix considering H-bond energies and highlight sidechain contact can yield the benefits to model the binding energy to gather statistics of the Ag-Ab interfaces.

### Large-scale peptide antigen identification on 389 pathogens

**Figure 5:**
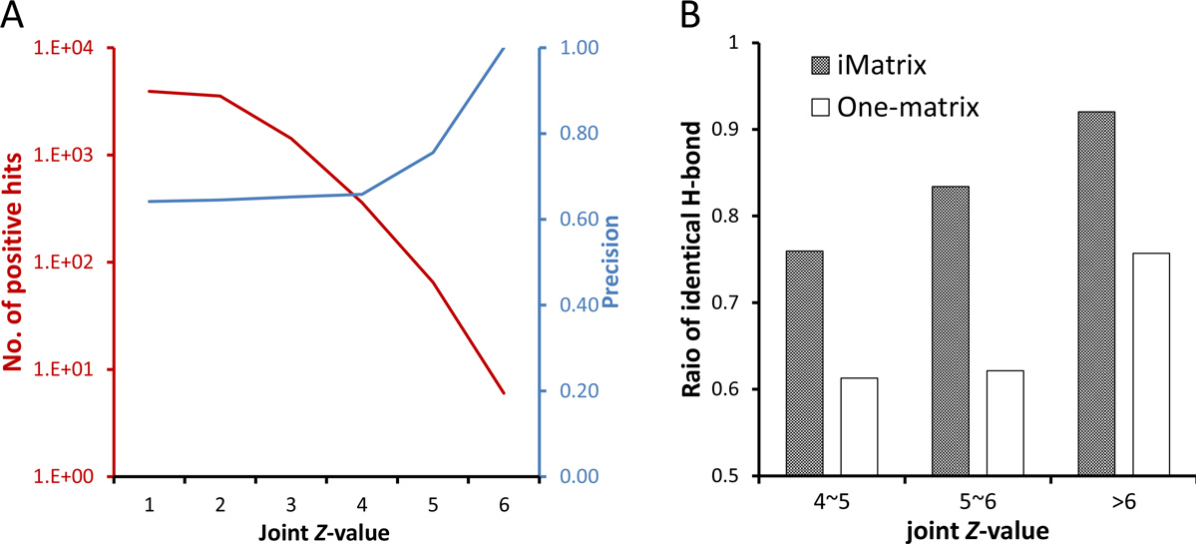
**The evaluation of iMatrix on 389 complete pathogen genome**. (A) Relationship between the positive hits (red line) and precision values (blue line) with different joint *Z*-value thresholds on 389 pathogens. (B) The ratios of the peptides with identical H-bond derived from the *i*Matrix are significantly increasing while the joint Z-value increases. The *i*Matrix outperforms the one-matrix.

To further investigate the reliability of *i*Matrix, we identified the homologous peptide antigens from 389 pathogens. Then, we collected 17 TCR-pMHC structure complexes (i.e. TCR-peptide-HLA-A0201) from PDB and 9,309 experimental peptide antigens (4,987 positive nonamers and 4,322 negative nonamers) from the IEDB [40] as the template, positive, and negative set, respectively. Among these pathogens, over 10^8 ^peptide candidates with *J_Z _*≥ 1.645 were selected for analyzing the relationships between *J_Z _*values with both the numbers of positive homologous peptide antigens (blue, recorded in IEDB) and precision (red). When *J_Z _*is higher than 4.0, the precision > 0.6 and the number of positive antigens exceeds 360 according to the positive and negative datasets (Figure 5A). If the *J_Z _*threshold is set to 4.0, the total number of inferring possible peptide antigens surpasses 700,000 statistically derived from 17 TCR-pMHC complexes. For 389 pathogens, we summarized the precision, the number of predicted homologous peptide antigens, and the positive and negative hits recorded in the IEDB for each pathogen (Table S5 in Additional file 7). Among these 389 pathogens, two *vaccinia viruses *have the most positive hits recorded in the IEDB and the precision of our method is higher than 0.65. Moreover, Table 1 shows the number of peptides (hits) in the peptide antigen families derived from the *i*Matrix and one-matrix. Although the precisions of homologous peptide antigen prediction have no difference under three different threshold (i.e. Joint *Z*-value ≥ 4, 5, and 6), the numbers of hits derived from *i*Matrix are significantly higher than derived from the one-matrix, especially while the threshold is set to 6 (Table 1). We also validated the peptide-immune recognitions in MHC alleles of H-2-Kb (*Mus musculus*) and H-2-Ld (*Mus musculus*) from the IEDB. The performance of *i*Matrix is consistently slightly superior to one matrix in three sets, HLA-A0201, H-2-Kb, and H-2-Ld (Table S6 in Additional file 8). These results implied that the homologous peptide antigen derived from *i*Matrix could achieve a better predicting accuracy.

**Table 1:** Comparisons between iMatrix and one-matrix on 389 complete pathogen database

**Joint** ***Z*-value**	***i*Matrix**				**One-matrix**				**(A)/(B)**
			
	No. of hits **(A)**	Positive hits	Negative hits	Precision	No. of hits **(B)**	Positive hits	Negative hits	Precision	
	
4	701,897	360	187	0.66	511,587	265	135	0.66	**1.37**
5	68,349	65	21	0.76	35,124	32	11	0.74	**1.95**
6	3,398	6	0	1	1,246	5	0	1	**2.73**

To further investigate the reliability of peptide compositions derived from difference matrices, we evaluated the hydrogen-bond (H-bond) ratio of each homologous peptide. The H-bond ratio is calculated as:

$$\text{H}-\text{bond}\;\text{ratio}=\frac{No.\;of\;H-bond\;with\;in\;the\;homologous\;peptide}{No.\;of\;H-bond\;within\;the\;template\;peptide}$$

where the H-bond ratio is equal to 1 while the number of H-bond within homologous peptide is equal to the template peptide (i.e. identical H-bond). Figure 5B illustrates the ratio of peptide which H-bond ratio equal to 1 within the peptide antigen family during different joint *Z*-value. The ratios of peptide with identical H-bond derived from the *i*Matrix have significant increasing while the threshold of joint *Z*-value is increasing. More importantly, the homologous peptides with joint *Z*-value > 6 derived from *i*Matrix have a significantly highest value of H-bond ratio (92%; Figure 5B). According our analysis described above, the H-bonds play an important role on the free energy of interface. Therefore, these peptide antigens with joint *Z*-value > 6 derived from *i*Matrix have a high precision value (Table 1) and keep the H-bond based on template (Figure 5) should be the more reliable peptide antigen than derived from one matrix.

### Homologous peptide antigens of Tax-1

**Figure 6:**
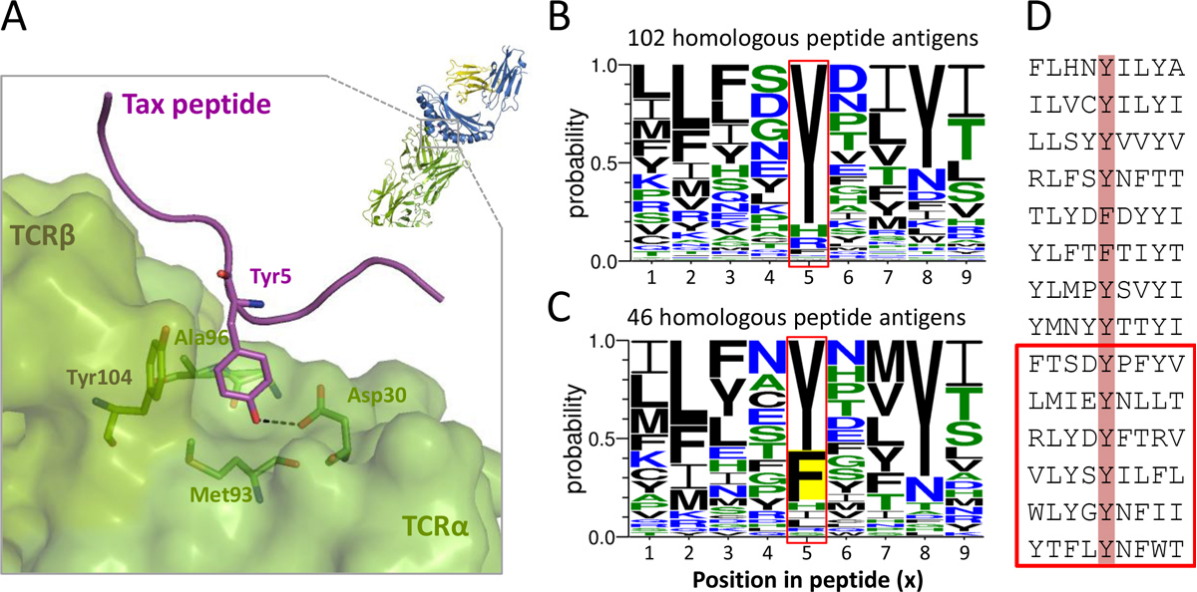
**Detailed binding model of TCR-peptide interface of template (i.e. Tax-1) and amino acid compositions of its corresponding peptide antigen families derived from iMatrix and one-matrix**. (A) Detailed binding model of TCR-peptide interface of Tax-1 (_11-19 _LLFGFPVYV of UniProt accession number: P0C213) by using the template (PDB entry: 1bd2). The amino acid composition (profiles) of the homologous peptide antigens derived from the (B) *i*Matrix and (C) one-matrix. (D) The 13 positive hits, recorded in the IEDB derived from *i*Matrix scoring function, are consensus (i.e. Tyr) in the position 5. The position 5 of 6 novel homologous peptides are the resdiue Tyr.

Protein Tax-1 is a transcriptional activator of *Human T-cell leukemia virus 1*(HTLV-1) [41]. The HTLV Tax protein is crucial for viral replication and for initiating malignant transformation leading to the development of adult T-cell leukemia [42]. Tax-1 has been shown to be oncogenic and also up-regulate interleukin 13 (IL-13), which is known to be linked to leukemogenesis [43]. The *i*Matrix scoring function can infer the experimental positive epitope of Tax-1 (_11-19 _LLFGFPVYV of UniProt [44] accession number: P0C213) and provides the detailed binding model based on its best hit template (PDB entry 1bd2 [45], Figure 6A). After Tax peptide (purplish cartoon in Figure 6A, residue 11-19 from Tax protein of HTLV-1) presented by extracellular domains of HLA-A0201, it is recognized by TCR (green region in Figure 6A) of *Homo sapiens*. The co-crystal TCR-pMHC structure assigned by *i*Matrix scoring function provides important contact residues and binding forces. Tyr5 of Tax peptide extends its aromatic sidechain deep into the pocket of TCR surface and forms one H-bond to Asp30 of TCR1α (black dash line in Figure 6A).

Furthermore, we would like to know whether the homologous peptide antigens of Tax peptide derived from *i*Matrix and one-matrix are different. The amino acid composition of the homologous peptide antigens was generated by by WebLogo, which is a graphical representation of an amino acid multiple sequence alignment [46]. The homologous peptide antigens originated in *i*Matrix are more than a double of the number originated in one-matrix (102 vs 46). The amino acid composition of the homologous peptide antigens *i*Matrix (Figure 6B) and one-matrix scoring function (Figure 6C) generating by WebLogo, which is a graphical representation of an amino acid multiple sequence alignment [46]. Two homologous peptide antigen sets maintained the important position 5 in peptide and conserved to Tyr (red frames in Figure 6B and 6C). This result conformed to the template-based atomic binding model (Figure 6A). Interestingly, position 5 in Figure 6B preferred all polar residues (Tyr, His, and Arg), whereas position 5 appeared Phe in Figure 6C (yellow background). However, Phe in position 5 of peptide is unreasonable and causes the loss of the critical H-bond. The *i*Matrix corrected such inaccuracy by considering special bond energies located in sidechain or backbone. Figure 2C provides the sidechain to sidechain special bond energies (*SFss_ij_*). According to the scores, Tyr to Asp is 7.3 (green box) and Phe to Asp is 0.0 (red box), respectively. These related results show the *i*Matrix reveals the interacting environment by individually evaluating binding force and locations.

The 13 positive hits which are recorded in the IEDB derived from *i*Matrix scoring function shows a high consensus in position 5 (red background in Figure 6D); moreover, position 5 of 6 novel homologous peptides (not discovered by one-matrix) in the red frame are exact to Tyr.

### Homologous peptide antigens of NY-ESO-1

**Figure 7:**
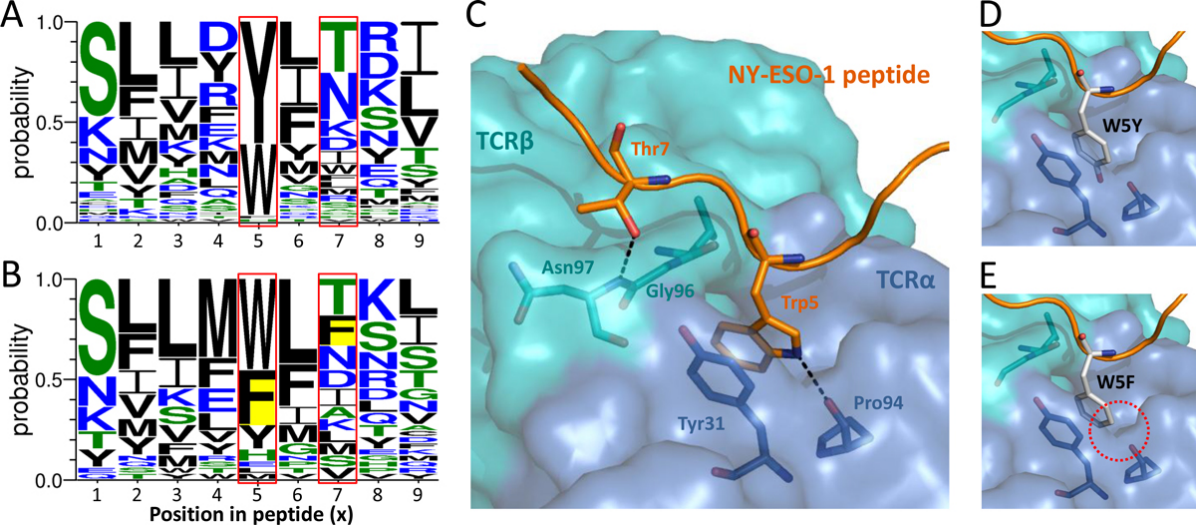
**Detailed binding model of TCR-peptide interface of template (i.e. NY-ESO-1)**. The amino acid compositions (profiles) of the homologous peptide antigens derived from the (A) *i*Matrix and (B) one-matrix. (C) Detailed binding model of TCR-peptide interface of NY-ESO-1 (157-165 SLLMWITQC) by using the template (PDB entry 2bnq). Specific point mutations (i.e. (D) W5Y and (E) W5F) on the position 5. The W5Y mutation in peptide and it reserves the H-bond and stacking interaction. However, the W5F mutation abolishes the hydrogen binding to TCR.

NY-ESO-1 is one of the most promising tumor-specific antigens, which was identified by the application of serological analysis of recombinant cDNA libraries from human tumors [47,48]. The *i*Matrix infers NY-ESO-1_157-165 _SLLMWITQC (UniProt accession number: P78358) on TCR recognition according to the structural template (PDB entry 2bnq [28]). The amino acid composition of homologous peptide antigens were generated by *i*Matrix (Figure 7A) and one-matrix (Figure 7B), respectively. According to *i*Matrix sensitive to atomic interactions between TCR and peptide, positions 5 and 7 of peptide (red frame) had particularly come into our notice that *i*Matrix excluded "Phe" from homologous peptide antigens in these two positions (yellow backgrund only showed in Figure 7B). The crystal structure demonstrated the rationality of *i*Matrix (Figure 7C). Trp5 of NY-ESO-1 peptide bound to Pro94 by forming one crucial H-bond and a stacking interaction with aromatic Tyr31 of TCRα; Thr7 has another important H-bond occurred in sidechain. We used PyMOL mutagenesis [49] to simulate the W5Y mutation in peptide and it might reserve the H-bond and stacking interaction (Figure 7D). These results corresponded with the position 5 of peptide where conserved to Trp and Tyr (Figure 7A); however, W5F mutation abolished hydrogen binding to TCR (circle in Figure 7E). As a result, the amino acid pattern suggested by *i*Matrix indeed revealed binding mechanism and maintained essential binding energy.

### Complementarity of interactions within a vdW network

**Figure 8:**
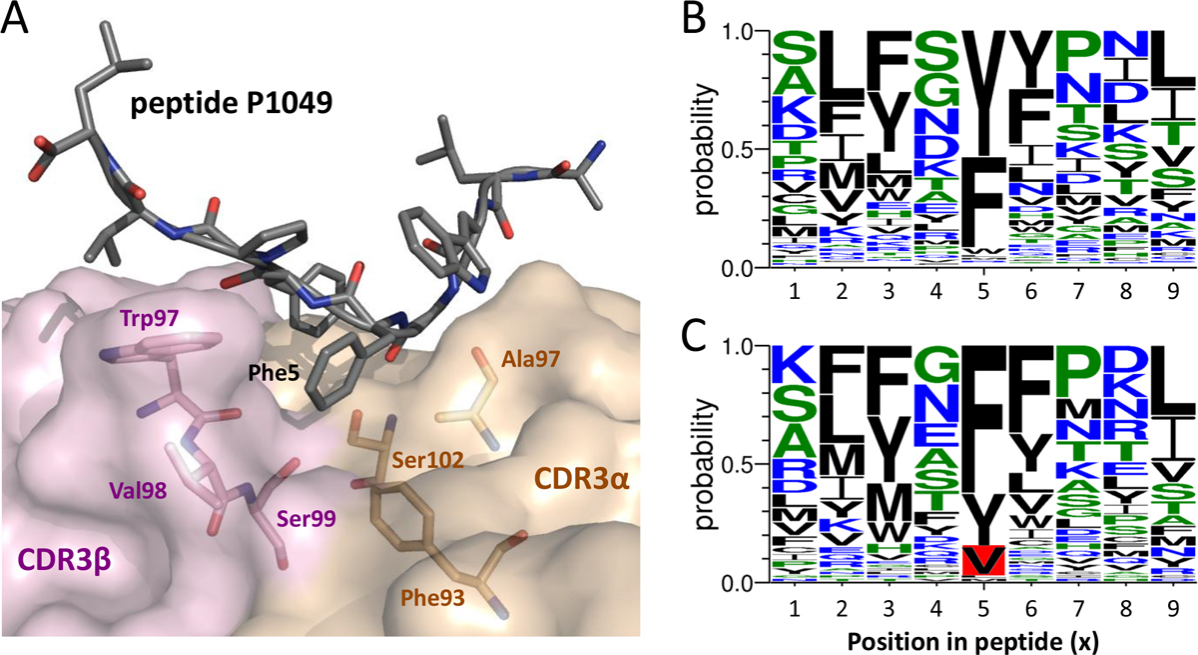
**Detailed binding model of TCR-peptide interface and amino acid composition of homologous peptide antigen derived from the template**. (A) Detailed binding model of TCR-peptide interface derived from the template (i.e. PDB entry: 1lp9). The amino acid compositions (profiles) of the homologous peptide antigens derived from the (B) *i*Matrix and (C) one-matrix. The position 5 in peptide prefer the aromatic residues (Phe, Tyr, and Trp) proposed by *i*Matrix. Therefore, the F5V is too small to stabilize van der Waals environments in the pocket.

*i*Matrix also evaluates binding environments abound with vdW forces well. Peptide P1049 appears to be stabilized in establishing a vdW network (Figure 8A) through Phe5 interacts with residues Phe93, Ala97, and Ser102 in the TCR CDR3α loop and Trp97, Val98, Ser99 in the TCR CDR3β loop (PDB entry 1lp9 [50] as template). *i*Matrix infers homologous peptide antigens drawn amino acid composition in Figure 8B and one-matrix's in Figure 8C. The position 5 in peptide has a preference for aromatic residues (Phe, Tyr, and Trp) proposed by *i*Matrix (Figure 8B); that is suitable for vdW environments in pocket. Val appeared in position 5 derived from one-matrix (red background in Figure 8C) is too small to stabilize the interface. In addition, ATYGVWPPV identified by using one-matrix is a negative epitope of *Vaccinia virus *recorded in the IEDB and could be filtered by *i*Matrix. The result implies that *i*Matrix performs van der Waals interactions with the sidechain contact modeling well than one-matrix.

## Conclusions

We have developed the *i*Matrix, PPI-scoring matrices and a template-based approach for modelling of TCR-pMHC interactions in a genome-wide scale. Our scoring matrices, including four knowledge-based scoring matrixes, are able to identify the significant hydrogen bonds and stacking interactions in the both TCR-peptide and MHC-peptide interfaces. Experimental results demonstrate that these matrices can yield high precisions of binding affinity and infer homologous peptide antigens of a template TCR-pMHC structure on 389 pathogen genomes. In addition, our structural TCR-pMHC models can provide detailed interacting models and crucial binding regions. We believe that our scoring matrixes and template-based method are able to provide biological insights and binding mechanisms of TCR-pMHC and to reveal the immune reactions for peptide vaccine designs.

## Competing interests

The authors declare that they have no competing interests.

## Authors' contributions

IHL, YSL, and JMY conceived and designed the experiments. IHL and YSL performed the experiments and drafted the manuscript; JMY supervised in the design of the study and helped to finalize the manuscript. All authors read and approved the final manuscript.

## Supplementary Material

Additional file 1 **One knowledge-based scoring matrix**. This matrix is a residue-based matrix derived from a non-redundant set which consists of 62 structural antigen-antibody complexes using in PAComplex Click here for file

Additional file 2 **Four knowledge-based protein-protein interacting scoring matrices**. The protein-protein scoring matrices consider sidechain-sidechain or sidechain-backbone vdW energies/special-bond energies in protein-protein interactions. Click here for file

Additional file 3 The 229 representative 3D structures of antibody-protein complexes derived from PDB Click here for file

Additional file 4 The 398 representative antigen-antibody interfaces for the generation of *i*Matrix Click here for file

Additional file 5 **The 70 mutated residues with free energy changes in 4 Ag-Ab interfaces**. The corresponding ΔΔG value indicates the change in free energy of binding upon mutation to alanine for each experimentally mutated residue derived from the ASEdb. Click here for file

Additional file 6 **The 17 TCR-peptide-HLA-A0201 complexes from the PDB**. This table contains PDB entry, chains of TCR, peptide, and HLA-A0201. Click here for file

Additional file 7 **The homologous peptide antigens in 389 pathogens with positive and negative hits recorded in the IEDB**. This table provides the precision, the number of predicted homologous peptide antigens, and the positive and negative hits recorded in the IEDB for 389 pathogens. Click here for file

Additional file 8 **Comparisons between *i*Matrix and one-matrix of three MHC class I alleles on 389 complete pathogen database**. Three MHC class I alleles are HLA-A0201 (*Homo sapiens*), H-2-Kb (*Mus musculus*), and H-2-Ld (*Mus musculus*). Click here for file

## Declarations

Publication of this article was funded by the "Program for Interdisciplinary Research Project in Bioinformatics" of National Science Council.

This article has been published as part of *BMC Genomics *Volume 14 Supplement 5, 2013: Twelfth International Conference on Bioinformatics (InCoB2013): Computational biology. The full contents of the supplement are available online at http://www.biomedcentral.com/bmcgenomics/supplements/14/S5.
