# Supplementary material for: Genome-wide structural modelling of TCR-pMHC interactions
Source: BMC Genomics. 2013 Oct 16;14(Suppl 5):S5. doi: 10.1186/1471-2164-14-S5-S5 (PMC3852114; doi:10.1186/1471-2164-14-S5-S5)
Supplement: Additional file 1 — One knowledge-based scoring matrix. This matrix is a residue-based matrix derived from a non-redundant set which consists of 62 structural antigen-antibody complexes using in PAComplex [file 1471-2164-14-S5-S5-S1.pdf]

	GLY	ALA	VAL	LEU	ILE	MET	PRO	PHE	TRP	TYR	CYS	SER	THR	ASN	GLN	HIS	ARG	LYS	GLU	ASP
**GLY**	-1.1	-1.1	-1.1	-1.0	-2.3	0.2	-0.9	0.1	-0.4	0.7	-0.3	-1.1	-0.1	-0.2	-0.2	-0.3	-0.3	-1.0	-0.1	-1.0
**ALA**	-1.1	-0.2	0.7	-2.0	-1.3	1.5	-1.3	2.0	0.3	1.1	1.3	-0.3	-0.4	0.1	-0.1	-0.7	-0.4	-2.4	-0.7	-2.1
**VAL**	-1.1	0.7	1.1	0.6	-1.9	-0.2	-2.5	1.4	1.2	0.6	0	-0.5	-0.3	-0.4	-1.6	-1.1	0.1	-3.1	-0.3	-2.5
**LEU**	-1.0	-2.0	0.6	-1.8	-0.6	-0.7	-0.3	0.6	0.7	1.2	0	-1.9	-1.5	-1.4	-1.1	-2.4	0.2	-1.8	-0.8	-1.1
**ILE**	-2.3	-1.3	-1.9	-0.6	0	1.4	-1.5	1.3	-0.5	1.3	0	-1.7	-0.8	-0.2	-0.4	-0.3	-0.5	-1.1	-2.9	-1.0
**MET**	0.2	1.5	-0.2	-0.7	1.4	1.7	1.2	0	1.6	0.7	0	-0.4	1.1	0.2	0	0.8	-0.7	0	-1.2	-2.8
**PRO**	-0.9	-1.3	-2.5	-0.3	-1.5	1.2	-2.9	1.0	1.5	1.0	0	-0.2	-0.6	-0.4	-1.4	1.1	-1.3	-2.1	-1.0	-1.9
**PHE**	0.1	2.0	1.4	0.6	1.3	0	1.0	2.3	-1.5	1.1	1.7	-0.1	-1.7	0.7	-0.8	-0.3	0.0	0.7	0.0	-0.3
**TRP**	-0.4	0.3	1.2	0.7	-0.5	1.6	1.5	-1.5	-0.7	0.1	1.0	0.1	-0.4	0.1	2.1	1.1	1.0	2.0	0.3	0.1
**TYR**	0.7	1.1	0.6	1.2	1.3	0.7	1.0	1.1	0.1	-1.2	-0.6	-0.1	-0.4	0.2	1.0	0.6	0.9	1.0	1.2	0.5
**CYS**	-0.3	1.3	0	0	0	0	0	1.7	1.0	-0.6	0	-2.0	-0.2	0.5	0	0.3	-1.3	0	0.0	0.2
**SER**	-1.1	-0.3	-0.5	-1.9	-1.7	-0.4	-0.2	-0.1	0.1	-0.1	-2.0	-0.7	-0.4	-0.2	0.6	-1.7	-0.4	-0.2	0.8	0.2
**THR**	-0.1	-0.4	-0.3	-1.5	-0.8	1.1	-0.6	-1.7	-0.4	-0.4	-0.2	-0.4	0.2	-0.6	0.5	-0.6	-0.7	-0.7	-0.3	-0.3
**ASN**	-0.2	0.1	-0.4	-1.4	-0.2	0.2	-0.4	0.7	0.1	0.2	0.5	-0.2	-0.6	0.5	0.7	0.6	0.2	-0.4	0.4	-0.3
**GLN**	-0.2	-0.1	-1.6	-1.1	-0.4	0	-1.4	-0.8	2.1	1.0	0	0.6	0.5	0.7	-2.7	0.3	-0.2	-1.2	-2.0	-0.6
**HIS**	-0.3	-0.7	-1.1	-2.4	-0.3	0.8	1.1	-0.3	1.1	0.6	0.3	-1.7	-0.6	0.6	0.3	-0.3	0.2	-0.1	0.1	0.1
**ARG**	-0.3	-0.4	0.1	0.2	-0.5	-0.7	-1.3	0.0	1.0	0.9	-1.3	-0.4	-0.7	0.2	-0.2	0.2	-0.4	-1.2	1.5	1.1
**LYS**	-1.0	-2.4	-3.1	-1.8	-1.1	0	-2.1	0.7	2.0	1.0	0	-0.2	-0.7	-0.4	-1.2	-0.1	-1.2	-1.9	0.8	1.2
**GLU**	-0.1	-0.7	-0.3	-0.8	-2.9	-1.2	-1.0	0.0	0.3	1.2	0	0.8	-0.3	0.4	-2.0	0.1	1.5	0.8	-2.6	-1.1
**ASP**	-1.0	-2.1	-2.5	-1.1	-1.0	-2.8	-1.9	-0.3	0.1	0.5	0.2	0.2	-0.3	-0.3	-0.6	0.1	1.1	1.2	-1.1	-1.4

Figure S1 - One knowledge-based scoring matrix in PAComplex

This matrix is a residue-based matrix derived from a non-redundant set which consists of 62 structural antigen-antibody complexes constructed by Ponomarenko *et al*. The interface prefers aromatic residues (i.e. Phe, Trp, and Tyr), which interact with aliphatic residues (i.e. Ala, Val, Leu, Ile, and Met) or long side-chain polar residues (i.e. Gln, His, Arg, Lys, and Glu), to form strong van der Waals forces (yellow boxes). Additionally, the scores are high if basic residues (i.e. Arg and Lys) interact with acidic residues (i.e. Asp and Glu). In contrast, the scores are low (purple box) when nonpolar residues interact to polar residues.
