# Supplementary figures and images for: Genome-wide structural modelling of TCR-pMHC interactions
Source: BMC Genomics. 2013 Oct 16;14(Suppl 5):S5. doi: 10.1186/1471-2164-14-S5-S5 (PMC3852114; doi:10.1186/1471-2164-14-S5-S5)

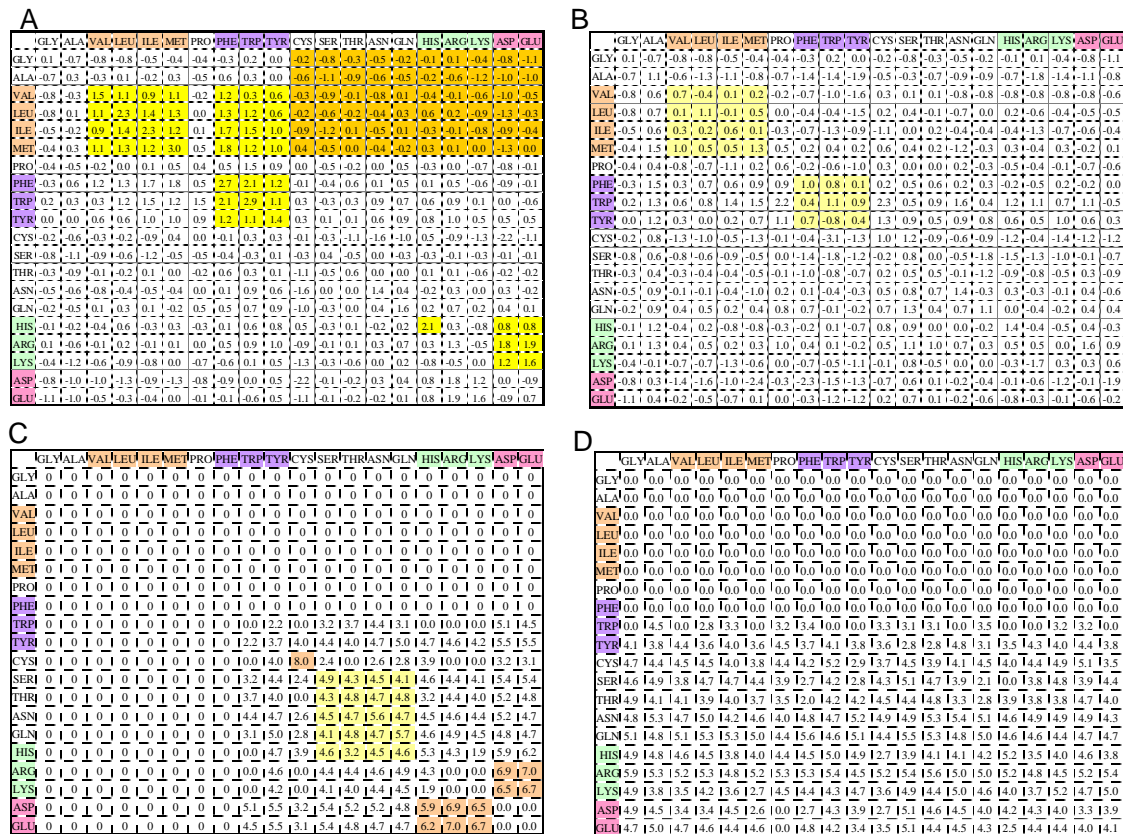

Supplement: Additional file 2 — Four knowledge-based protein-protein interacting scoring matrices. The protein-protein scoring matrices consider sidechain-sidechain or sidechain-backbone vdW energies/special-bond energies in protein-protein interactions. [file 1471-2164-14-S5-S5-S2.pdf]
