# Supplementary material for: Genome-wide structural modelling of TCR-pMHC interactions
Source: BMC Genomics. 2013 Oct 16;14(Suppl 5):S5. doi: 10.1186/1471-2164-14-S5-S5 (PMC3852114; doi:10.1186/1471-2164-14-S5-S5)
Supplement: Additional file 3 — The 229 representative 3D structures of antibody-protein complexes derived from PDB [file 1471-2164-14-S5-S5-S3.pdf]

Table S1 - The 229 representative 3D structures of antibody-protein complexes derived from PDB with high resolution (≤3Å)

Tag	PDB entry	IMGT molecule name	IMGT receptor description	Species	Experimental technique	Resolution
1	1a3r	8F5	FAB-GAMMA-2A_KAPPA	Mus musculus	X-ray diffraction	2.1
2	1acy	59.1 mAb, anti-gp120 [HIV-1]	FAB-GAMMA-1_KAPPA	Mus musculus	X-ray diffraction	3
3	1ahw	5G9	FAB-GAMMA-1_KAPPA	Mus musculus	X-ray diffraction	3
4	1ar1	1AR1	FV-HEAVY_KAPPA	Mus musculus	X-ray diffraction	2.7
5	1bj1	Bevacizumab Fab, 12-IgG1 Fab, F(ab)-12 IgG1, Fab-12 IgG1, rhuMAb-VEGF Fab, AVASTINR	FAB-GAMMA-1_KAPPA	Humanized	X-ray diffraction	2.4
6	1bog	Cb41	FAB-GAMMA-2C_KAPPA	Mus musculus	X-ray diffraction	2.6
7	1bql	HyHEL-5	FAB-GAMMA-1_KAPPA	Mus musculus	X-ray diffraction	2.6
8	1cfn	Cb41 mAb, anti-p24 [HIV-1]	FAB-GAMMA-2C_KAPPA	Mus musculus	X-ray diffraction	2.65
9	1cfs	Cb41 mAb, anti-p24 [HIV-1]	FAB-GAMMA-2C_KAPPA	Mus musculus	X-ray diffraction	2.75
10	1cu4	3F4	FAB-GAMMA-2A_KAPPA	Mus musculus	X-ray diffraction	2.9
11	1cz8	Ranibizumab, Fab-12 variant Y0317, LUCENTISR	FAB-GAMMA-1_KAPPA	Humanized	X-ray diffraction	2.4
12	1dn2	FcG1-2	FC-GAMMA-1	Homo sapiens	X-ray diffraction	2.7
13	1dzb	1F9	SCFV-HEAVY-KAPPA	Mus musculus	X-ray diffraction	2
14	1e6j	13B5 mAb, anti-p24 [HIV-1]	FAB-GAMMA-1_KAPPA	Mus musculus	X-ray diffraction	3
15	1egj	Bion-1	FAB-GAMMA-2A_KAPPA	Mus musculus	X-ray diffraction	2.8
16	1ejo	4C4	FAB-GAMMA-2A_KAPPA	Mus musculus	X-ray diffraction	2.3
17	1eo8	Bh151	FAB-GAMMA-1_KAPPA	Mus musculus	X-ray diffraction	2.8
18	1f58	58.2 mAb, anti-gp120 [HIV-1]	FAB-GAMMA-1_KAPPA	Mus musculus	X-ray diffraction	2
19	1fbi	F9.13.7	FAB-GAMMA-1_KAPPA	Mus musculus	X-ray diffraction	3
20	1fc2	IG Gamma-1 Chain C Region	(CH2-CH3)	Homo sapiens	X-ray diffraction	2.8
21	1fdl	D1.3 mAb, anti-lysozyme C [Chicken]	FAB-GAMMA-1_KAPPA	Mus musculus	X-ray diffraction	2.5
22	1fe8	Ru5	FAB-GAMMA-2A_KAPPA	Mus musculus	X-ray diffraction	2.03
23	1fj1	La2	FAB-GAMMA-2B_KAPPA	Mus musculus	X-ray diffraction	2.68
24	1frg	41178	FAB-GAMMA-2A_KAPPA	Mus musculus	X-ray diffraction	2.8
25	1fsk	Mst2 / Bv16	FAB-GAMMA-1_KAPPA	Mus musculus	X-ray diffraction	2.9
26	1ggi	50.1 mAb, anti-gp120 [HIV-1]	FAB-GAMMA-2A_KAPPA	Mus musculus	X-ray diffraction	2.8
27	1h0d	26-2F neutralizing mAb, anti-angiogenin (ANG) [human]	FAB-GAMMA-1_KAPPA	Mus musculus	X-ray diffraction	2
28	1hez	Fab 2A2	FAB-MU_KAPPA	Homo sapiens	X-ray diffraction	2.7
29	1hh9	Cb41 mAb, anti-p24 [HIV-1]	FAB-GAMMA-2C_KAPPA	Mus musculus	X-ray diffraction	2.7
30	1hi6	Cb41 mAb, anti-p24 [HIV-1]	FAB-GAMMA-2C_KAPPA	Mus musculus	X-ray diffraction	2.55
31	1i8k	Epidermal Growth Factor Receptor Antibody	FV-HEAVY_KAPPA	Mus musculus	X-ray diffraction	1.8
32	1igc	MOPC21	FAB-GAMMA-1_KAPPA	Mus musculus	X-ray diffraction	2.6
33	1ikf	R45-45-11 CsA specific Fab	FAB-GAMMA-1_KAPPA	Mus musculus	X-ray diffraction	2.5

34	1iqd	BO2C11	FAB-GAMMA-4_KAPPA	Homo sapiens	X-ray diffraction	2
35	1jhl	D11.15	FV-HEAVY_KAPPA	Mus musculus	X-ray diffraction	2.4
36	1jps	D3H44	FAB-GAMMA-1_KAPPA	Homo sapiens	X-ray diffraction	1.85
37	1jrh	A6	FAB-GAMMA-1_KAPPA	Mus musculus	X-ray diffraction	2.8
38	1kb5	Desire-1;KB5-C20	FAB-GAMMA-2A_KAPPA;FV-ALPHA_BETA	Mus musculus	X-ray diffraction	2.5
39	1kb9	Fv-1	FV-HEAVY_KAPPA	Mus musculus	X-ray diffraction	2.3
40	1kc5	PC287	FAB-GAMMA-1_KAPPA	Mus musculus	X-ray diffraction	2.5
41	1kcr	PC283	FAB-GAMMA-1_KAPPA	Mus musculus	X-ray diffraction	2.9
42	1kcs	PC282	FAB-GAMMA-1_KAPPA	Mus musculus	X-ray diffraction	2.5
43	1ktr	Anti-His Tag Antibody 3D5 Variable Heavy Chain	FV-HEAVY_KAPPA	Mus musculus	X-ray diffraction	2.7
44	1kxq	CABAMD9	(VH)	Camelus dromedarius	X-ray diffraction	1.6
45	1kxt	Immunoglobulin Vhh Fragment	(VH)	Camelus dromedarius	X-ray diffraction	2
46	1kyo	Fv-1	FV-HEAVY_KAPPA	Mus musculus	X-ray diffraction	2.97
47	1l6x	Rituximab, CH2-CH3 of H-GAMMA-1, RITUXANR, MABTHERAR	(CH2-CH3)	Chimeric	X-ray diffraction	1.65
48	1lk3	9D7	FAB-GAMMA-1_KAPPA	Rattus norvegicus	X-ray diffraction	1.91
49	1mel	Cab-LYS3	(VH)	Camelus dromedarius	X-ray diffraction	2.5
50	1mhp	INTEGRIN-ALPHA-1;huAQC2	(I-DOMAIN);FAB-GAMMA-1_KAPPA	Chimeric;Humanized	X-ray diffraction	2.8
51	1mvf	Immunoglobulin Heavy Chain Variable Region	(VH)	Camelus dromedarius	X-ray diffraction	1.65
52	1n0x	B12 neutralizing mAb, anti-gp120 [HIV-1]	FAB-GAMMA-1_KAPPA	Homo sapiens	X-ray diffraction	1.8
53	1n64	19D9D6	FAB-GAMMA-1_KAPPA	Mus musculus	X-ray diffraction	2.34
54	1n6q	Fab 28	FAB-GAMMA-1_KAPPA	Mus musculus	X-ray diffraction	3
55	1n8z	Trastuzumab Fab, 4D5-humanized variant 8 Fab, Herceptin Fab, HERCEPTINR	FAB-GAMMA-1_KAPPA	Humanized	X-ray diffraction	2.52
56	1nak	83.1	FAB-GAMMA-1_KAPPA	Mus musculus	X-ray diffraction	2.57
57	1nby	Anti-Lysozyme Antibody HyHEL-63	FAB-GAMMA-2A_KAPPA	Mus musculus	X-ray diffraction	1.8
58	1nca	NC41	FAB-GAMMA-2A_KAPPA	Mus musculus	X-ray diffraction	2.5
59	1ncb	NC41	FAB-GAMMA-2A_KAPPA	Mus musculus	X-ray diffraction	2.5
60	1ndg	Anti-Lysozyme Antibody HyHEL-8	FAB-GAMMA-2A_KAPPA	Mus musculus	X-ray diffraction	1.9
61	1nl0	Anti-factor IX antibody 10C12 Fab	FAB-GAMMA-1_LAMBDA	Homo sapiens	X-ray diffraction	2.2
62	1nma	NC10	FV-HEAVY_KAPPA	Mus musculus	X-ray diffraction	3
63	1nsn	N10	FAB-GAMMA-1_KAPPA	Mus musculus	X-ray diffraction	2.9
64	1oak	Nmc-4	FAB-GAMMA-1_KAPPA	Mus musculus	X-ray diffraction	2.2
65	1oaz	SPE-7 Fv, anti-2,4-dinitrophenyl (DNP) IgE antibody SPE-7	FV-HEAVY_LAMBDA	Mus musculus	X-ray diffraction	2.77
66	1ob1	Antibody	FAB-GAMMA-2A_KAPPA	Mus musculus	X-ray diffraction	2.9
67	1ors	33H1	FAB-GAMMA-1_KAPPA	Mus musculus	X-ray diffraction	1.9
68	1osp	184.1	FAB-GAMMA-2B_KAPPA	Mus musculus	X-ray diffraction	1.95
69	1ots	Antibody Fab Fragment	FAB-GAMMA-1_KAPPA	Mus musculus	X-ray diffraction	2.51
70	1p2c	F10.6.6	FAB-GAMMA-1_KAPPA	Mus musculus	X-ray diffraction	2
71	1p4b	Fv fragment	FV-HEAVY_LAMBDA	Mus musculus	X-ray diffraction	2.35

72	1pkq	MOG;8-18C5	1V-LIKE;FAB-GAMMA-1_KAPPA	Rattus norvegicus;Chimeric	X-ray diffraction	3
73	1q1j	447-52D	FAB-GAMMA-3_LAMBDA	Homo sapiens	X-ray diffraction	2.5
74	1qfu	Influenza hemagglutinin neutralizing Fab	FAB-GAMMA-1_KAPPA	Mus musculus	X-ray diffraction	2.8
75	1qkz	MN14C11.6	FAB-GAMMA-2A_KAPPA	Mus musculus	X-ray diffraction	1.95
76	1r3i	Antibody Fab Fragment	FAB-GAMMA-1_KAPPA	Mus musculus	X-ray diffraction	2.4
77	1ri8	1D2L19	(VH)	Camelus dromedarius	X-ray diffraction	1.85
78	1rjc	CAB-LYS2	(VH)	Camelus dromedarius	X-ray diffraction	1.4
79	1rjl	Fab H6831	FAB-GAMMA-2A_KAPPA	Mus musculus	X-ray diffraction	2.6
80	1sm3	Sm3	FAB-GAMMA-1_LAMBDA	Mus musculus	X-ray diffraction	1.95
81	1sy6	OKT3;CD3G_CD3E	FAB-GAMMA-2A_KAPPA;2C-LIKE	Mus musculus;Homo sapiens	X-ray diffraction	2.1
82	1tet	Te33	FAB-GAMMA-1_KAPPA	Mus musculus	X-ray diffraction	2.3
83	1tji	2F5 neutralizing mAb, anti-gp41 [HIV-1]	FAB-GAMMA-1_KAPPA	Homo sapiens	X-ray diffraction	2.2
84	1tpx	VRQ14	FAB-GAMMA-2A_KAPPA	Mus musculus	X-ray diffraction	2.56
85	1tzg	40000000000	FAB-GAMMA-1_KAPPA	Homo sapiens	X-ray diffraction	2.2
86	1tzh	YADS1	FAB-GAMMA-1_KAPPA	Mus musculus	X-ray diffraction	2.6
87	1tzi	Fab YADS2	FAB-GAMMA-1_KAPPA	Mus musculus	X-ray diffraction	2.8
88	1uac	Anti-hen lysozyme Fv HyHEL-10 SFSF	FV-HEAVY_KAPPA	Mus musculus	X-ray diffraction	1.7
89	1uj3	HATR-5	FAB-GAMMA-4_KAPPA	Homo sapiens	X-ray diffraction	2.1
90	1v7m	TN1	FAB-GAMMA-1_KAPPA	Mus musculus	X-ray diffraction	2.51
91	1w72	HLA-A*0101;HYB3	MH1-ALPHA_B2M;FAB-GAMMA-1_LAMBDA	Homo sapiens	X-ray diffraction	2.15
92	1wej	E8	FAB-GAMMA-1_KAPPA	Mus musculus	X-ray diffraction	1.8
93	1yjd	CD28;5.11A1	1V-LIKE;FAB-GAMMA-1_KAPPA	Homo sapiens;Mus musculus	X-ray diffraction	2.7
94	1ymh	Fab 19D9D6 anti-HCV	FAB-GAMMA-1_KAPPA	Mus musculus	X-ray diffraction	2.6
95	1yy9	Cetuximab, IMC-C225, ERBITUXR	FAB-GAMMA-1_KAPPA	Chimeric	X-ray diffraction	2.6
96	1yyl	17B mAb, anti-gp120 [HIV-1]	FAB-GAMMA-1_KAPPA	Homo sapiens	X-ray diffraction	2.75
97	1ztx	E16	FAB-GAMMA-2B_KAPPA	Mus musculus	X-ray diffraction	2.5
98	1zv5	D2-L29	(VH)	Camelus dromedarius	X-ray diffraction	2
99	1zvh	D2-L24	(VH)	Camelus dromedarius	X-ray diffraction	1.5
100	1zvy	D3-L11	(VH)	Camelus dromedarius	X-ray diffraction	1.63
101	2a6i	36-65	FAB-GAMMA-1_KAPPA	Mus musculus	X-ray diffraction	2.5
102	2adf	82D6A3	FAB-GAMMA-2A_KAPPA	Mus musculus	X-ray diffraction	1.9
103	2aep	Mem5	FAB-GAMMA-2A_KAPPA	Mus musculus	X-ray diffraction	2.1
104	2ap2	C219	FV-HEAVY_KAPPA	Mus musculus	X-ray diffraction	2.4
105	2arj	YTS 105.18;CD8A_CD8A homodimer	FAB-GAMMA-2B_KAPPA;1V-LIKE_1V-LIKE	Rattus norvegicus;Mus musculus	X-ray diffraction	2.88

106	2b1a	HIV-1 neutralizing Fab 2219	FAB-GAMMA-1_LAMBDA	Homo sapiens	X-ray diffraction	2.35
107	2b1h	Anti-HIV-1 V3 Fab 2219	FAB-GAMMA-1_LAMBDA	Homo sapiens	X-ray diffraction	2
108	2b2x	AQC2 Fab, S28>Q, T50>V, N52>Y, K64>E;Integrin alpha-1	FAB-GAMMA-1_KAPPA;(INTEGRIN-ALPHA-1)	Mus musculus;Rattus norvegicus	X-ray diffraction	2.2
109	2bdn	11K2	FAB-GAMMA-2A_KAPPA	Mus musculus	X-ray diffraction	2.53
110	2brr	MN20B9.34	FAB-GAMMA-2A_KAPPA	Mus musculus	X-ray diffraction	1.95
111	2bse	Llama heavy chain VH domain	(VH)	Lama glama	X-ray diffraction	2.7
112	2ck0	Anti-anti-idiotypic antibody against Human angiotensin II Fab 131	FAB-GAMMA-1_KAPPA	Mus musculus	X-ray diffraction	2.2
113	2dd8	Fab M396	FAB-GAMMA-1_LAMBDA	Homo sapiens	X-ray diffraction	2.3
114	2dqc	HyHEL-10 mutant, Y33>F	FV-HEAVY_KAPPA	Mus musculus	X-ray diffraction	1.8
115	2dwd	Antibody Fab Fragment	FAB-GAMMA-1_KAPPA	Mus musculus	X-ray diffraction	2.6
116	2f58	58.2 mAb, anti-gp120 [HIV-1]	FAB-GAMMA-1_KAPPA	Mus musculus	X-ray diffraction	2.8
117	2fd6	ATN-615	FAB-GAMMA-1_KAPPA	Mus musculus	X-ray diffraction	1.9
118	2fjg	G6	FAB-GAMMA-1_KAPPA	Homo sapiens	X-ray diffraction	2.8
119	2ghw	Anti-sars scFv 80R	SCFV-HEAVY-KAPPA	Homo sapiens	X-ray diffraction	2.3
120	2hmi	28 mAb, anti-reverse transcriptase [HIV-1]	FAB-GAMMA-1_KAPPA	Mus musculus	X-ray diffraction	2.8
121	2hrp	F11.2.32 mAb, anti-protease [HIV-1]	FAB-GAMMA-1_KAPPA	Mus musculus	X-ray diffraction	2.2
122	2iff	HyHEL-5	FAB-GAMMA-1_KAPPA	Mus musculus	X-ray diffraction	2.65
123	2igf	B13I2	FAB-GAMMA-1_KAPPA	Mus musculus	X-ray diffraction	2.8
124	2iwg	FcG1-5	FC-GAMMA-1	Homo sapiens	X-ray diffraction	2.35
125	2j4w	F8.12.19	FAB-GAMMA-1_KAPPA	Mus musculus	X-ray diffraction	2.5
126	2j5l	F8.12.19	FAB-GAMMA-1_KAPPA	Mus musculus	X-ray diffraction	2.9
127	2jel	JEL42	FAB-GAMMA-1_KAPPA	Mus musculus	X-ray diffraction	2.5
128	2ny7	B12 Fab, anti-gp120 [HIV-1]	FAB-GAMMA-1_KAPPA	Homo sapiens	X-ray diffraction	2.3
129	2osl	Rituximab, IDEC-C2B8, RITUXANR, MABTHERAR	FAB-GAMMA-1_KAPPA	Chimeric	X-ray diffraction	2.6
130	2p4a	Cab-RN05	(VH)	Camelus dromedarius	X-ray diffraction	1.9
131	2r56	IgE Fab Fragment	FAB-GAMMA-1_KAPPA	Homo sapiens	X-ray diffraction	2.8
132	2vdk	1000000;INTEGRIN-ALPHA-IIB_BETA-3	FAB-GAMMA-2C_KAPPA;INTEGRIN-ALPHA-IIB_BETA-3	Mus musculus;Homo sapiens	X-ray diffraction	2.8
133	2vol	IG Gamma-1 Chain C Region	(CH2-CH3)	Mus musculus	X-ray diffraction	1.95
134	2vxq	IgE Fab Fragment	FAB-GAMMA-1_KAPPA	Homo sapiens	X-ray diffraction	1.9
135	2vxs	Antibody Fab Fragment	FAB-GAMMA-1_LAMBDA	Homo sapiens	X-ray diffraction	2.63
136	2vxt	125-2H Fab	FAB-GAMMA-1_KAPPA	Mus musculus	X-ray diffraction	1.49
137	2vyr	Single VH domain	(VH)	Homo sapiens	X-ray diffraction	2
138	2w9e	Anti-PRP Fab ICSM 18	FAB-GAMMA-1_KAPPA	Mus musculus	X-ray diffraction	2.9
139	2wuc	Fab 40.DELTATRP	FAB-GAMMA-1_KAPPA	Homo sapiens	X-ray diffraction	2.7
140	2xqb	DISC0280 Fab, anti-IL-15	FAB-GAMMA-1_LAMBDA	Homo sapiens	X-ray diffraction	2.6
141	2xqy	Fab A13-D6.3 mAb	FAB-GAMMA-2A_KAPPA	Mus musculus	X-ray diffraction	2.05

142	2xra	Fab HK20 mAb, anti-gp41 [HIV-1]	FAB-GAMMA-1_KAPPA	Homo sapiens	X-ray diffraction	2.3
143	2xtj	Fab 1D05	FAB-GAMMA-1_KAPPA	Homo sapiens	X-ray diffraction	2.7
144	2xwt	Fab K1-70, monoclonal autoantibody	FAB-GAMMA-1_LAMBDA	Homo sapiens	X-ray diffraction	1.9
145	2xzq	Fab BBE6.12H3, anti-(4-hydroxy-3-nitrophenyl)-acetyl (NP)	FAB-GAMMA-1_LAMBDA	Mus musculus	X-ray diffraction	2.4
146	2y06	Fab BBE6.12H3, anti-(4-hydroxy-3-nitrophenyl)-acetyl (NP)	FAB-GAMMA-1_LAMBDA	Mus musculus	X-ray diffraction	2.5
147	2y07	Fab BBE6.12H3, anti-(4-hydroxy-3-nitrophenyl)-acetyl (NP)	FAB-GAMMA-1_LAMBDA	Mus musculus	X-ray diffraction	2.4
148	2y36	Fab BBE6.12H3, anti-(4-hydroxy-3-nitrophenyl)-acetyl (NP)	FAB-GAMMA-1_LAMBDA	Mus musculus	X-ray diffraction	2.7
149	2y6s	Fab 14G7 mAb, anti-envelope glycoprotein (GP) mucin-like domain [Ebola virus]	FAB-GAMMA-1_KAPPA	Mus musculus	X-ray diffraction	2.8
150	2yc1	9004G scFv	SCFV-HEAVY-KAPPA	Homo sapiens	X-ray diffraction	1.9
151	2znw	ScFv10	SCFV-KAPPA-HEAVY	Homo sapiens	X-ray diffraction	2.71
152	3bae	WO2 pepAbeta(1-28), anti-Abeta peptide	FAB-GAMMA-2A_KAPPA	Mus musculus	X-ray diffraction	1.59
153	3be1	BH1	FAB-GAMMA-1_KAPPA	Homo sapiens	X-ray diffraction	2.9
154	3bgf	Fab F26G19	FAB-GAMMA-2C_KAPPA	Mus musculus	X-ray diffraction	3
155	3bn9	E2	FAB-GAMMA-1_KAPPA	Homo sapiens	X-ray diffraction	2.17
156	3c2a	447-52D neutralizing mAb, anti-gp120 [HIV-1]	FAB-GAMMA-3_LAMBDA	Homo sapiens	X-ray diffraction	2.1
157	3cvh	25-D1.16;H2-K1b	FAB-GAMMA-1_KAPPA;MH1-ALPHA_B2M	Mus musculus	X-ray diffraction	2.9
158	3cxd	Anti-osteopontin antibody 23C3	FAB-GAMMA-1_KAPPA	Mus musculus	X-ray diffraction	2.8
159	3d0l	2F5 neutralizing mAb, anti-gp41 [HIV-1]	FAB-GAMMA-1_KAPPA	Homo sapiens	X-ray diffraction	2.35
160	3d85	7G10 Fab	FAB-GAMMA-1_KAPPA	Chimeric	X-ray diffraction	1.9
161	3dsf	Anti-osteopontin antibody 23C3	FAB-GAMMA-1_KAPPA	Mus musculus	X-ray diffraction	2.8
162	3dus	SAG506-01	FV-HEAVY_KAPPA	Mus musculus	X-ray diffraction	1.95
163	3dvg	K63-specific Fab APU.3A8	FAB-GAMMA-1_KAPPA	Homo sapiens	X-ray diffraction	2.6
164	3e8u	Fab 106.3 mAb	FAB-GAMMA-1_KAPPA	Mus musculus	X-ray diffraction	2.1
165	3eoa	Integrin alpha-L;Efalizumab Fab fragment	(INTEGRIN-ALPHA-L);FAB-GAMMA-1_KAPPA	Homo sapiens	X-ray diffraction	2.8
166	3ffd	Monoclonal antibody Fab	FAB-GAMMA-1_LAMBDA	Mus musculus	X-ray diffraction	2
167	3g04	Thyroid-stimulating human monoclonal autoantibody (M22)	FAB-GAMMA-1_LAMBDA	Homo sapiens	X-ray diffraction	2.55
168	3g5v	Fab 806, monoclonal Antibody against EGFR	FAB-GAMMA-2B_KAPPA	Mus musculus	X-ray diffraction	2
169	3gbm	Fab CR6261	FAB-GAMMA-1_LAMBDA	Homo sapiens	X-ray diffraction	2.7
170	3gbn	Fab CR6261	FAB-GAMMA-1_LAMBDA	Homo sapiens	X-ray diffraction	2.2
171	3ghb	Fab 447-52D mAb, anti-HIV-1	FAB-GAMMA-3_LAMBDA	Homo sapiens	X-ray diffraction	2.25
172	3ghe	Fab 537-10D mAb, anti-HIV-1	FAB-GAMMA-3_LAMBDA	Homo sapiens	X-ray diffraction	2.4
173	3gi8	7F11 mAb, anti-ApcT	FAB-GAMMA-2B_KAPPA	Mus musculus	X-ray diffraction	2.59
174	3gjf	HLA-A*0201;Antibody Fab	MH1-ALPHA_B2M;FAB-GAMMA-1_LAMBDA	Homo sapiens	X-ray diffraction	1.9
175	3go1	Fab 268-D anti-HIV-1	FAB-GAMMA-1_LAMBDA	Homo sapiens	X-ray diffraction	1.89
176	3h0t	Antibody Fab Fragment	FAB-GAMMA-1_LAMBDA	Homo sapiens	X-ray diffraction	1.89

177	3h3b	Anti-ErbB2 antibody chA21 scFv	SCFV-KAPPA-HEAVY	Mus musculus	X-ray diffraction	2.45
178	3h3p	4E10 (H)W104>A mAb, anti-HIV-1	FV-HEAVY_KAPPA	Homo sapiens	X-ray diffraction	2.7
179	3hae	Antibody Fab;HLA-A*0201	FAB-GAMMA-1_LAMBDA;MH1-ALPHA_B2M	Homo sapiens	X-ray diffraction	2.9
180	3hi6	Fab AL-57;Integrin alpha-L (LFA-1A), L186>C, F324>C	FAB-GAMMA-4_KAPPA;(INTEGRIN-ALPHA-L)	Homo sapiens	X-ray diffraction	2.3
181	3hr5	Quilizumab, 47H4, Anti-M1', MEMP1972A, RG-7449	FAB-GAMMA-1_KAPPA	Humanized	X-ray diffraction	2.4
182	3idx	B13 neutralizing mAb, anti-gp120 [HIV-1]	FAB-GAMMA-1_KAPPA	Homo sapiens	X-ray diffraction	2.5
183	3ifn	Fab 12A11	FAB-GAMMA-1_KAPPA	Mus musculus	X-ray diffraction	1.5
184	3iu3	Basiliximab, basiliximab Fab, SimulectR	FAB-GAMMA-1_KAPPA	Chimeric	X-ray diffraction	2.9
185	3jwd	Fab 48D;CD4	FAB-GAMMA-1_KAPPA;1V-1C-LIKE	Homo sapiens	X-ray diffraction	2.61
186	3k7u	Llama heavy chain VH domain	(VH)	Lama glama	X-ray diffraction	2.1
187	3k80	Llama heavy chain VH domain	(VH)	Lama glama	X-ray diffraction	2.4
188	3kr3	DX-2647 mAb, anti-IGF-II	FAB-GAMMA-1_KAPPA	Homo sapiens	X-ray diffraction	2.2
189	3l5w	Anti-human IL-13 antibody C836 Fab	FAB-GAMMA-1_KAPPA	Homo sapiens	X-ray diffraction	2
190	3l95	Anti-NRR1 Fab	FAB-GAMMA-1_KAPPA	Homo sapiens	X-ray diffraction	2.19
191	3ldb	Antibody Fab Fragment	FAB-GAMMA-1_KAPPA	Rattus norvegicus	X-ray diffraction	2.7
192	3lev	2F5 neutralizing mAb, anti-gp41 [HIV-1]	FAB-GAMMA-1_KAPPA	Homo sapiens	X-ray diffraction	2.5
193	3lh2	4E10 Fv, W104>A	FV-HEAVY_KAPPA	Homo sapiens	X-ray diffraction	2.65
194	3lhp	4E10 Fv, W104>A	FV-HEAVY_KAPPA	Homo sapiens	X-ray diffraction	2.7
195	3liz	Fab 4C3 mAb	(L-KAPPA_VH-CH1-HINGE-REGION)	Mus musculus	X-ray diffraction	1.8
196	3lzf	2D1 Fab	FAB-GAMMA-1_LAMBDA	Homo sapiens	X-ray diffraction	2.8
197	3mlr	Fab 2557 mAb anti-HIV-1	FAB-GAMMA-1_LAMBDA	Homo sapiens	X-ray diffraction	1.8
198	3mls	Fab 2557 mAb anti-HIV-1	FAB-GAMMA-1_LAMBDA	Homo sapiens	X-ray diffraction	2.5
199	3mlt	Fab 2557 mAb anti-HIV-1	FAB-GAMMA-1_LAMBDA	Homo sapiens	X-ray diffraction	2.49
200	3mlu	Fab 2557 mAb anti-HIV-1	FAB-GAMMA-1_LAMBDA	Homo sapiens	X-ray diffraction	2.77
201	3mlv	Fab 2557 mAb anti-HIV-1	FAB-GAMMA-1_LAMBDA	Homo sapiens	X-ray diffraction	2.48
202	3mlw	Fab 1006-15D mAb anti-HIV-1	FAB-GAMMA-1_LAMBDA	Homo sapiens	X-ray diffraction	2.7
203	3mlx	Fab 3074 mAb anti-HIV-1	FAB-GAMMA-1_LAMBDA	Homo sapiens	X-ray diffraction	1.9
204	3mly	Fab 3074 mAb anti-HIV-1	FAB-GAMMA-1_LAMBDA	Homo sapiens	X-ray diffraction	1.7
205	3mlz	Fab 3074 mAb anti-HIV-1	FAB-GAMMA-1_LAMBDA	Homo sapiens	X-ray diffraction	2.99
206	3mnw	Fab 13H11 mAb, anti-HIV-1	FAB-GAMMA-1_KAPPA	Chimeric	X-ray diffraction	2.2
207	3mxw	5E1 Fab	FAB-GAMMA-1_KAPPA	Homo sapiens	X-ray diffraction	1.83
208	3ngb	Fab VRC01 mAb, anti-HIV-1	FAB-GAMMA-1_KAPPA	Homo sapiens	X-ray diffraction	2.68
209	3nh7	Fab ABD1556	FAB-GAMMA-1_LAMBDA	Homo sapiens	X-ray diffraction	2.7
210	3nps	Fab S4	(V-LAMBDA-C-KAPPA_VH-CH1)	Homo sapiens	X-ray diffraction	1.5
211	3o2d	T-cell surface glycoprotein CD4 (P01730);ibalizumab Fab, anti-CD4	1V-1C-LIKE;FAB-GAMMA-4_KAPPA	Homo sapiens;Humanized	X-ray diffraction	2.19
212	3o41	Fab 101F mAb	FAB-GAMMA-1_KAPPA	Mus musculus	X-ray diffraction	1.95

213	3o45	Fab 101F mAb	FAB-GAMMA-1_KAPPA	Mus musculus	X-ray diffraction	2.87
214	3pgf	Synthetic antigen binder (sAB)	FAB-GAMMA-1_KAPPA	Homo sapiens	X-ray diffraction	2.1
215	3pp4	Obinutuzumab, GA101 Fab	FAB-GAMMA-1_KAPPA	Humanized	X-ray diffraction	1.6
216	3qwo	Motavizumab Fab	FAB-GAMMA-1_KAPPA	Mus musculus	X-ray diffraction	1.9
217	3r1g	YW412.8.31 Fab	FAB-GAMMA-1_KAPPA	Homo sapiens	X-ray diffraction	2.8
218	3rvv	Fab 4C1 mAb, Der f 1 dust mite allergen	FAB-GAMMA-1_KAPPA	Mus musculus	X-ray diffraction	1.9
219	3rvw	Fab 4C1 mAb, Der p 1 dust mite allergen	FAB-GAMMA-1_KAPPA	Mus musculus	X-ray diffraction	1.95
220	3s35	Fab 6.64, anti-VEGF receptor 2;Vascular endothelial growth factor receptor 2 (P35968)	FAB-GAMMA-1_KAPPA;(KDR(Pr225-325_d3,326-327))	Homo sapiens	X-ray diffraction	2.2
221	3s37	Vascular endothelial growth factor receptor 2 (P35968);Fab 1121B, anti-VEGF receptor 2	(KDR(Pr225-325_d3,326-327));FAB-GAMMA-1_KAPPA	Homo sapiens	X-ray diffraction	2.7
222	3sdy	Fab CR8020	FAB-GAMMA-1_KAPPA	Homo sapiens	X-ray diffraction	2.85
223	3se8	Fab VRC03	FAB-GAMMA-1_KAPPA	Homo sapiens	X-ray diffraction	1.9
224	3skj	Fab anti-human EphA2	FAB-GAMMA-1_KAPPA	Homo sapiens	X-ray diffraction	2.5
225	3sob	Antibody Fab	FAB-GAMMA-1_KAPPA	Homo sapiens	X-ray diffraction	1.9
226	3t2n	Fab hH35 mAb, anti-HPN (hepsin), a type II transmembrane serine protease	FAB-GAMMA-1_KAPPA	Homo sapiens	X-ray diffraction	2.55
227	3u0t	Fab ponezumab anti-amyloid beta (A beta)	FAB-GAMMA-2_KAPPA	Humanized	X-ray diffraction	2.5
228	3u30	FAB	FAB-GAMMA-1_KAPPA	Homo sapiens	X-ray diffraction	2.43
229	3ztn	Fab FI6V3	FAB-GAMMA-1_KAPPA	Homo sapiens	X-ray diffraction	3
