# Supplementary material for: Genome-wide structural modelling of TCR-pMHC interactions
Source: BMC Genomics. 2013 Oct 16;14(Suppl 5):S5. doi: 10.1186/1471-2164-14-S5-S5 (PMC3852114; doi:10.1186/1471-2164-14-S5-S5)
Supplement: Additional file 4 — The 398 representative antigen-antibody interfaces for the generation of iMatrix [file 1471-2164-14-S5-S5-S4.pdf]

Table S2 - The 398 representative antigen-antibody interfaces for the generation of *i*Matrix

Tag	PDB	Ag-Ab interface	Protein 1	Protein 2	Molecule of Protein 1	Molecule of Protein 2
1	1a3r	1a3rHP	1a3r_H	1a3r_P	IGG2A 8F5 FAB (HEAVY CHAIN)	HUMAN RHINOVIRUS CAPSID PROTEIN VP2
2	1a3r	1a3rLP	1a3r_L	1a3r_P	IGG2A 8F5 FAB (LIGHT CHAIN)	HUMAN RHINOVIRUS CAPSID PROTEIN VP2
3	1acy	1acyHP	1acy_H	1acy_P	IGG1-KAPPA 59.1 FAB (HEAVY CHAIN)	HIV-1 GP120 (MN ISOLATE)
4	1acy	1acyLP	1acy_L	1acy_P	IGG1-KAPPA 59.1 FAB (LIGHT CHAIN)	HIV-1 GP120 (MN ISOLATE)
5	1ahw	1ahwDF	1ahw_D	1ahw_F	IMMUNOGLOBULIN FAB 5G9 (LIGHT CHAIN)	TISSUE FACTOR
6	1ahw	1ahwEF	1ahw_E	1ahw_F	IMMUNOGLOBULIN FAB 5G9 (HEAVY CHAIN)	TISSUE FACTOR
7	1ar1	1ar1BC	1ar1_B	1ar1_C	CYTOCHROME C OXIDASE	ANTIBODY FV FRAGMENT
8	1ar1	1ar1BD	1ar1_B	1ar1_D	CYTOCHROME C OXIDASE	ANTIBODY FV FRAGMENT
9	1bj1	1bj1KV	1bj1_K	1bj1_V	FAB FRAGMENT	VASCULAR ENDOTHELIAL GROWTH FACTOR
10	1bog	1bogAC	1bog_A	1bog_C	ANTIBODY (CB 4-1)	PEPTIDE
11	1bog	1bogBC	1bog_B	1bog_C	ANTIBODY (CB 4-1)	PEPTIDE
12	1bql	1bqlHY	1bql_H	1bql_Y	HYHEL-5 FAB (HEAVY CHAIN)	BOBWHITE QUAIL LYSOZYME
13	1cfn	1cfnAC	1cfn_A	1cfn_C	PROTEIN (IGG2A KAPPA ANTIBODY CB41 (LIGHT CHA	PROTEIN (BOUND PEPTIDE)
14	1cfn	1cfnBC	1cfn_B	1cfn_C	PROTEIN (IGG2A-KAPPA ANTIBODY CB41 (HEAVY CHA	PROTEIN (BOUND PEPTIDE)
15	1cfs	1cfsAC	1cfs_A	1cfs_C	PROTEIN (IGG2A KAPPA ANTIBODY CB41 (LIGHT CHA	PROTEIN (ANTIGEN BOUND PEPTIDE)
16	1cfs	1cfsBC	1cfs_B	1cfs_C	PROTEIN (IGG2A KAPPA ANTIBODY CB41 (HEAVY CHA	PROTEIN (ANTIGEN BOUND PEPTIDE)
17	1cu4	1cu4HP	1cu4_H	1cu4_P	FAB HEAVY CHAIN	RECOGNITION PEPTIDE
18	1cu4	1cu4LP	1cu4_L	1cu4_P	FAB LIGHT CHAIN	RECOGNITION PEPTIDE
19	1cz8	1cz8VX	1cz8_V	1cz8_X	VASCULAR ENDOTHELIAL GROWTH FACTOR	LIGHT CHAIN OF NEUTRALIZING ANTIBODY
20	1dn2	1dn2AE	1dn2_A	1dn2_E	IMMUNOGLOBULIN LAMBDA HEAVY CHAIN	ENGINEERED PEPTIDE
21	1dzb	1dzbAX	1dzb_A	1dzb_X	SCFV FRAGMENT 1F9	TURKEY EGG-WHITE LYSOZYME C
22	1e6j	1e6jHP	1e6j_H	1e6j_P	IMMUNOGLOBULIN	CAPSID PROTEIN P24
23	1e6j	1e6jLP	1e6j_L	1e6j_P	IMMUNOGLOBULIN	CAPSID PROTEIN P24
24	1egj	1egjAH	1egj_A	1egj_H	CYTOKINE RECEPTOR COMMON BETA CHAIN PRECURSOR	ANTIBODY (HEAVY CHAIN)
25	1egj	1egjAL	1egj_A	1egj_L	CYTOKINE RECEPTOR COMMON BETA CHAIN PRECURSOR	ANTIBODY (LIGHT CHAIN)
26	1ejo	1ejoHP	1ejo_H	1ejo_P	IGG2A MONOCLONAL ANTIBODY (HEAVY CHAIN)	FMDV PEPTIDE
27	1ejo	1ejoLP	1ejo_L	1ejo_P	IGG2A MONOCLONAL ANTIBODY (LIGHT CHAIN)	FMDV PEPTIDE
28	1eo8	1eo8AH	1eo8_A	1eo8_H	HEMAGGLUTININ (HA1 CHAIN)	ANTIBODY (HEAVY CHAIN)
29	1eo8	1eo8AL	1eo8_A	1eo8_L	HEMAGGLUTININ (HA1 CHAIN)	ANTIBODY (LIGHT CHAIN)
30	1f58	1f58LP	1f58_L	1f58_P	PROTEIN (IGG1 ANTIBODY 58.2 (LIGHT CHAIN))	PROTEIN (EXTERIOR MEMBRANE GLYCOPROTEIN(GP120
31	1fbi	1fbiHX	1fbi_H	1fbi_X	IGG1 F9.13.7 FAB (HEAVY CHAIN)	GUINEA FOWL LYSOZYME
32	1fbi	1fbiLX	1fbi_L	1fbi_X	IGG1 F9.13.7 FAB (LIGHT CHAIN)	GUINEA FOWL LYSOZYME
33	1fc2	1fc2CD	1fc2_C	1fc2_D	FRAGMENT B OF PROTEIN A COMPLEX	IMMUNOGLOBULIN FC
34	1fdl	1fdlHY	1fdl_H	1fdl_Y	IGG1-KAPPA D1.3 FAB (HEAVY CHAIN)	HEN EGG WHITE LYSOZYME
35	1fdl	1fdlLY	1fdl_L	1fdl_Y	IGG1-KAPPA D1.3 FAB (LIGHT CHAIN)	HEN EGG WHITE LYSOZYME
36	1fe8	1fe8AH	1fe8_A	1fe8_H	VON WILLEBRAND FACTOR	IMMUNOGLOBULIN IGG RU5

37	1fe8	1fe8AL	1fe8_A	1fe8_L	VON WILLEBRAND FACTOR	IMMUNOGLOBULIN IGG RU5
38	1fj1	1fj1AF	1fj1_A	1fj1_F	HYBRIDOMA ANTIBODY LA2 (LIGHT CHAIN)	OUTER SURFACE PROTEIN A
39	1fj1	1fj1BF	1fj1_B	1fj1_F	HYBRIDOMA ANTIBODY LA2 (HEAVY CHAIN)	OUTER SURFACE PROTEIN A
40	1frg	1frgHP	1frg_H	1frg_P	IGG2A 26/9 FAB (HEAVY CHAIN)	INFLUENZA HEMAGGLUTININ HA1 (STRAIN X47) (RES
41	1frg	1frgLP	1frg_L	1frg_P	IGG2A 26/9 FAB (LIGHT CHAIN)	INFLUENZA HEMAGGLUTININ HA1 (STRAIN X47) (RES
42	1fsk	1fskAB	1fsk_A	1fsk_B	MAJOR POLLEN ALLERGEN BET V 1-A	IMMUNOGLOBULIN KAPPA LIGHT CHAIN
43	1fsk	1fskAC	1fsk_A	1fsk_C	MAJOR POLLEN ALLERGEN BET V 1-A	ANTIBODY HEAVY CHAIN FAB
44	1ggi	1ggiHP	1ggi_H	1ggi_P	IGG2A 50.1 FAB (HEAVY CHAIN)	HIV-1 V3 LOOP PEPTIDE ANTIGEN
45	1ggi	1ggiLP	1ggi_L	1ggi_P	IGG2A 50.1 FAB (LIGHT CHAIN)	HIV-1 V3 LOOP PEPTIDE ANTIGEN
46	1h0d	1h0dAC	1h0d_A	1h0d_C	ANTIBODY FAB FRAGMENT, LIGHT CHAIN	ANGIOGENIN
47	1h0d	1h0dBC	1h0d_B	1h0d_C	ANTIBODY FAB FRAGMENT, HEAVY CHAIN	ANGIOGENIN
48	1hez	1hezAE	1hez_A	1hez_E	KAPPA LIGHT CHAIN OF IG	PROTEIN L
49	1hez	1hezCE	1hez_C	1hez_E	KAPPA LIGHT CHAIN OF IG	PROTEIN L
50	1hh9	1hh9BC	1hh9_B	1hh9_C	IGG2A KAPPA ANTIBODY CB41 (HEAVY CHAIN)	PEP-2
51	1hi6	1hi6AC	1hi6_A	1hi6_C	IGG2A KAPPA ANTIBODY CB41 (LIGHT CHAIN)	PEPTIDE 5
52	1hi6	1hi6BC	1hi6_B	1hi6_C	IGG2A KAPPA ANTIBODY CB41 (HEAVY CHAIN)	PEPTIDE 5
53	1i8k	1i8kAC	1i8k_A	1i8k_C	EPIDERMAL GROWTH FACTOR RECEPTOR ANTIBODY MR1	EPIDERMAL GROWTH FACTOR RECEPTOR, EGFRVIII PE
54	1i8k	1i8kBC	1i8k_B	1i8k_C	EPIDERMAL GROWTH FACTOR RECEPTOR ANTIBODY MR1	EPIDERMAL GROWTH FACTOR RECEPTOR, EGFRVIII PE
55	1igc	1igcAH	1igc_A	1igc_H	STREPTOCOCCAL PROTEIN G (DOMAIN III)	IGG1-KAPPA MOPC21 FAB (HEAVY CHAIN)
56	1igc	1igcAL	1igc_A	1igc_L	STREPTOCOCCAL PROTEIN G (DOMAIN III)	IGG1-KAPPA MOPC21 FAB (LIGHT CHAIN)
57	1ikf	1ikfCH	1ikf_C	1ikf_H	CYCLOSPORIN A	IGG1-KAPPA R45-45-11 FAB (HEAVY CHAIN)
58	1iqd	1iqdAC	1iqd_A	1iqd_C	HUMAN MONOCLONAL BO2C11 FAB LIGHT CHAIN	HUMAN FACTOR VIII
59	1iqd	1iqdBC	1iqd_B	1iqd_C	HUMAN MONOCLONAL BO2C11 FAB HEAVY CHAIN	HUMAN FACTOR VIII
60	1jhl	1jhlAH	1jhl_A	1jhl_H	PHEASANT EGG WHITE LYSOZYME	IGG1-KAPPA D11.15 FV (HEAVY CHAIN)
61	1jhl	1jhlAL	1jhl_A	1jhl_L	PHEASANT EGG WHITE LYSOZYME	IGG1-KAPPA D11.15 FV (LIGHT CHAIN)
62	1jps	1jpsHT	1jps_H	1jps_T	Immunoglobulin Fab D3H44, heavy chain	Tissue factor
63	1jrh	1jrhHI	1jrh_H	1jrh_I	ANTIBODY A6	INTERFERON-GAMMA RECEPTOR ALPHA CHAIN
64	1jrh	1jrhIL	1jrh_I	1jrh_L	INTERFERON-GAMMA RECEPTOR ALPHA CHAIN	ANTIBODY A6
65	1kb5	1kb5AH	1kb5_A	1kb5_H	KB5-C20 T-CELL ANTIGEN RECEPTOR	ANTIBODY DESIRE-1
66	1kb5	1kb5AL	1kb5_A	1kb5_L	KB5-C20 T-CELL ANTIGEN RECEPTOR	ANTIBODY DESIRE-1
67	1kb5	1kb5BH	1kb5_B	1kb5_H	KB5-C20 T-CELL ANTIGEN RECEPTOR	ANTIBODY DESIRE-1
68	1kb5	1kb5BL	1kb5_B	1kb5_L	KB5-C20 T-CELL ANTIGEN RECEPTOR	ANTIBODY DESIRE-1
69	1kb9	1kb9EK	1kb9_E	1kb9_K	UBIQUINOL-CYTOCHROME C REDUCTASE IRON-SULFUR	LIGHT CHAIN (VL) OF FV-FRAGMENT
70	1kc5	1kc5HP	1kc5_H	1kc5_P	PC287 Immunoglobulin	PS1 peptide
71	1kcr	1kcrHP	1kcr_H	1kcr_P	PC283 IMMUNOGLOBULIN	PS1 peptide
72	1kcr	1kcrLP	1kcr_L	1kcr_P	PC283 IMMUNOGLOBULIN	PS1 peptide
73	1kcs	1kcsLP	1kcs_L	1kcs_P	PC282 IMMUNOGLOBULIN	PS1 peptide
74	1ktr	1ktrLM	1ktr_L	1ktr_M	Anti-his tag antibody 3d5 variable light chai	Peptide linker
75	1kxq	1kxqAH	1kxq_A	1kxq_H	Alpha-amylase, pancreatic	antibody VHH fragment CABAMD9

76	1kxt	1kxtAB	1kxt_A	1kxt_B	ALPHA-AMYLASE, PANCREATIC	IMMUNOGLOBULIN VHH FRAGMENT
77	1kyo	1kyoPU	1kyo_P	1kyo_U	UBIQUINOL-CYTOCHROME C REDUCTASE IRON-SULFUR	HEAVY CHAIN (VH) OF FV-FRAGMENT
78	1l6x	1l6xAB	1l6x_A	1l6x_B	IMMUNOGLOBULIN GAMMA-1 HEAVY CHAIN CONSTANT R	Minimized B-domain of Protein A Z34C
79	1lk3	1lk3AH	1lk3_A	1lk3_H	Interleukin-10	9D7 Heavy Chain
80	1lk3	1lk3AL	1lk3_A	1lk3_L	Interleukin-10	9D7 Light Chain
81	1mel	1melAL	1mel_A	1mel_L	VH SINGLE-DOMAIN ANTIBODY	LYSOZYME
82	1mhp	1mhpAL	1mhp_A	1mhp_L	Integrin alpha 1, (RESIDUES 169-360)	FAB FRAGMENT, light chain
83	1mvf	1mvfAE	1mvf_A	1mvf_E	Immunoglobulin heavy chain variable region	PemI-like protein 1
84	1n0x	1n0xHP	1n0x_H	1n0x_P	IMMUNOGLOBULIN HEAVY CHAIN	B2.1 peptide
85	1n0x	1n0xLP	1n0x_L	1n0x_P	IMMUNOGLOBULIN LIGHT CHAIN	B2.1 peptide
86	1n64	1n64HP	1n64_H	1n64_P	Fab 19D9D6 heavy chain	Genome polyprotein Capsid protein C
87	1n64	1n64LP	1n64_L	1n64_P	Fab 19D9D6 light chain	Genome polyprotein Capsid protein C
88	1n6q	1n6qBH	1n6q_B	1n6q_H	Reverse Transcriptase	Monoclonal Antibody (Heavy Chain)
89	1n6q	1n6qBL	1n6q_B	1n6q_L	Reverse Transcriptase	Monoclonal Antibody (Light Chain)
90	1n8z	1n8zAC	1n8z_A	1n8z_C	Herceptin Fab (antibody) - light chain	Receptor protein-tyrosine kinase erbB-2
91	1n8z	1n8zBC	1n8z_B	1n8z_C	Herceptin Fab (antibody) - heavy chain	Receptor protein-tyrosine kinase erbB-2
92	1nak	1nakHP	1nak_H	1nak_P	Fab 83.1 - heavy chain	Peptide MP1
93	1nak	1nakLP	1nak_L	1nak_P	Fab 83.1 - light chain	Peptide MP1
94	1nby	1nbyAC	1nby_A	1nby_C	Antibody kappa light chain	Lysozyme C
95	1nca	1ncaLN	1nca_L	1nca_N	IGG2A-KAPPA NC41 FAB (LIGHT CHAIN)	INFLUENZA A SUBTYPE N9 NEURAMINIDASE
96	1ncb	1ncbHN	1ncb_H	1ncb_N	IGG2A-KAPPA NC41 FAB (HEAVY CHAIN)	INFLUENZA A SUBTYPE N9 NEURAMINIDASE
97	1ndg	1ndgBC	1ndg_B	1ndg_C	Immunoglobulin gamma 1 chain	Lysozyme C
98	1nl0	1nl0GH	1nl0_G	1nl0_H	Factor IX	Anti-factor IX antibody, 10C12, chain H
99	1nl0	1nl0GL	1nl0_G	1nl0_L	Factor IX	Anti-factor IX antibody, 10C12, chain L
100	1nma	1nmaHN	1nma_H	1nma_N	FAB NC10	N9 NEURAMINIDASE
101	1nma	1nmaLN	1nma_L	1nma_N	FAB NC10	N9 NEURAMINIDASE
102	1nsn	1nsnHS	1nsn_H	1nsn_S	IGG FAB (IGG1, KAPPA)	STAPHYLOCOCCAL NUCLEASE
103	1nsn	1nsnLS	1nsn_L	1nsn_S	IGG FAB (IGG1, KAPPA)	STAPHYLOCOCCAL NUCLEASE
104	1oak	1oakAH	1oak_A	1oak_H	VON WILLEBRAND FACTOR	NMC-4 IGG1
105	1oak	1oakAL	1oak_A	1oak_L	VON WILLEBRAND FACTOR	NMC-4 IGG1
106	1oaz	1oazAH	1oaz_A	1oaz_H	THIOREDOXIN 1	IMMUNOGLOBULIN E
107	1oaz	1oazAL	1oaz_A	1oaz_L	THIOREDOXIN 1	IMMUNOGLOBULIN E
108	1ob1	1ob1AC	1ob1_A	1ob1_C	ANTIBODY, HEAVY CHAIN	MAJOR MEROZOITE SURFACE PROTEIN
109	1ob1	1ob1BC	1ob1_B	1ob1_C	ANTIBODY, LIGHT CHAIN	MAJOR MEROZOITE SURFACE PROTEIN
110	1ors	1orsAC	1ors_A	1ors_C	33H1 Fab light chain	Potassium channel
111	1ors	1orsBC	1ors_B	1ors_C	33H1 Fab heavy chain	Potassium channel
112	1osp	1ospHO	1osp_H	1osp_O	FAB 184.1	OUTER SURFACE PROTEIN A
113	1osp	1ospLO	1osp_L	1osp_O	FAB 184.1	OUTER SURFACE PROTEIN A
114	1ots	1otsBE	1ots_B	1ots_E	Voltage-gated ClC-type chloride channel eriC	Fab fragment (heavy chain)

115	1ots	1otsBF	1ots_B	1ots_F	Voltage-gated ClC-type chloride channel eriC	Fab fragment (light chain)
116	1p2c	1p2cAC	1p2c_A	1p2c_C	light chain anti-lysozyme antibody F10.6.6	Lysozyme C
117	1p2c	1p2cBC	1p2c_B	1p2c_C	heavy chain VH+CH1 anti-lysozyme antibody F10	Lysozyme C
118	1p4b	1p4bHP	1p4b_H	1p4b_P	Antibody Variable heavy chain	GCN4(7P-14P) peptide
119	1p4b	1p4bLP	1p4b_L	1p4b_P	Antibody Variable light chain	GCN4(7P-14P) peptide
120	1pkq	1pkqAE	1pkq_A	1pkq_E	(8-18C5) chimeric Fab, light chain	Myelin Oligodendrocyte Glycoprotein
121	1pkq	1pkqBE	1pkq_B	1pkq_E	(8-18C5) chimeric Fab, heavy chain	Myelin Oligodendrocyte Glycoprotein
122	1q1j	1q1jLP	1q1j_L	1q1j_P	Fab 447-52D, light chain	Gp120 V3 peptide
123	1qfu	1qfuAH	1qfu_A	1qfu_H	PROTEIN (HEMAGGLUTININ (HA1 CHAIN))	PROTEIN (IMMUNOGLOBULIN IGG1-KAPPA ANTIBODY (
124	1qfu	1qfuAL	1qfu_A	1qfu_L	PROTEIN (HEMAGGLUTININ (HA1 CHAIN))	PROTEIN (IMMUNOGLOBULIN IGG1-KAPPA ANTIBODY (
125	1qkz	1qkzHP	1qkz_H	1qkz_P	ANTIBODY	MAJOR OUTER MEMBRANE PROTEIN P1.16
126	1qkz	1qkzLP	1qkz_L	1qkz_P	ANTIBODY	MAJOR OUTER MEMBRANE PROTEIN P1.16
127	1r3i	1r3iCH	1r3i_C	1r3i_H	Voltage-gated potassium channel	Antibody Fab fragment heavy chain
128	1ri8	1ri8AB	1ri8_A	1ri8_B	camelid ANTIBODY HEAVY CHAIN	Lysozyme C
129	1rjc	1rjcAB	1rjc_A	1rjc_B	camelid heavy chain antibody	Lysozyme C
130	1rjl	1rjlAC	1rjl_A	1rjl_C	Fab H6831 L-chain	Outer surface protein B
131	1rjl	1rjlBC	1rjl_B	1rjl_C	Fab H6831 H-chain	Outer surface protein B
132	1sm3	1sm3HP	1sm3_H	1sm3_P	SM3 ANTIBODY	PEPTIDE EPITOPE
133	1sm3	1sm3LP	1sm3_L	1sm3_P	SM3 ANTIBODY	PEPTIDE EPITOPE
134	1sy6	1sy6AH	1sy6_A	1sy6_H	T-cell surface glycoprotein CD3 gamma/epsilon	OKT3 Fab heavy chain
135	1sy6	1sy6AL	1sy6_A	1sy6_L	T-cell surface glycoprotein CD3 gamma/epsilon	OKT3 Fab light chain
136	1tet	1tetHP	1tet_H	1tet_P	IGG1 TE33 FAB (HEAVY CHAIN)	CHOLERA TOXIN PEPTIDE 3 (CTP3)
137	1tet	1tetLP	1tet_L	1tet_P	IGG1 TE33 FAB (LIGHT CHAIN)	CHOLERA TOXIN PEPTIDE 3 (CTP3)
138	1tji	1tjiHP	1tji_H	1tji_P	Anti-HIV-1 antibody 2F5 Heavy Chain	Envelope Glycoprotein GP41
139	1tji	1tjiLP	1tji_L	1tji_P	Anti-HIV-1 antibody 2F5 Light Chain	Envelope Glycoprotein GP41
140	1tpx	1tpxAB	1tpx_A	1tpx_B	Major prion protein	The VRQ14 Fab
141	1tpx	1tpxAC	1tpx_A	1tpx_C	Major prion protein	The VRQ14 Fab
142	1tzg	1tzgHP	1tzg_H	1tzg_P	Fab 4E10	Envelope polyprotein GP160
143	1tzh	1tzhAW	1tzh_A	1tzh_W	Fab YADS1 Light Chain	Vascular endothelial growth factor A
144	1tzh	1tzhBW	1tzh_B	1tzh_W	Fab YADS1 Heavy Chain	Vascular endothelial growth factor A
145	1tzi	1tziAV	1tzi_A	1tzi_V	Fab YADS2 Light Chain	Vascular endothelial growth factor A
146	1tzi	1tziBV	1tzi_B	1tzi_V	Fab YADS2 Heavy Chain	Vascular endothelial growth factor A
147	1uac	1uacHY	1uac_H	1uac_Y	Ig VH,anti-lysozyme	Lysozyme C
148	1uj3	1uj3AC	1uj3_A	1uj3_C	IgG Fab light chain	tissue factor
149	1uj3	1uj3BC	1uj3_B	1uj3_C	IgG Fab heavy chain	tissue factor
150	1v7m	1v7mHV	1v7m_H	1v7m_V	Monoclonal TN1 Fab Heavy Chain	Thrombopoietin
151	1v7m	1v7mLV	1v7m_L	1v7m_V	Monoclonal TN1 Fab Light Chain	Thrombopoietin
152	1w72	1w72AH	1w72_A	1w72_H	HLA CLASS I HISTOCOMPATIBILITY ANTIGEN	HYB3 HEAVY CHAIN
153	1w72	1w72AL	1w72_A	1w72_L	HLA CLASS I HISTOCOMPATIBILITY ANTIGEN	HYB3 LIGHT CHAIN

154	1wej	1wejFH	1wej_F	1wej_H	CYTOCHROME C	E8 ANTIBODY
155	1wej	1wejFL	1wej_F	1wej_L	CYTOCHROME C	E8 ANTIBODY
156	1yjd	1yjdCH	1yjd_C	1yjd_H	T-cell-specific surface glycoprotein CD28	Fab fragment of 5.11A1 antibody heavy chain
157	1yjd	1yjdCL	1yjd_C	1yjd_L	T-cell-specific surface glycoprotein CD28	Fab fragment of 5.11A1 antibody light chain
158	1ymh	1ymhAE	1ymh_A	1ymh_E	Fab 16D9D6, light chain	Protein L
159	1ymh	1ymhBE	1ymh_B	1ymh_E	Fab 16D9D6, heavy chain	Protein L
160	1yy9	1yy9AC	1yy9_A	1yy9_C	Epidermal Growth Factor Receptor	Cetuximab Fab Light chain
161	1yy9	1yy9AD	1yy9_A	1yy9_D	Epidermal Growth Factor Receptor	Cetuximab Fab Heavy chain
162	1yyl	1yylGH	1yyl_G	1yyl_H	Exterior membrane glycoprotein(GP120)	Antibody 17b heavy chain
163	1yyl	1yylGL	1yyl_G	1yyl_L	Exterior membrane glycoprotein(GP120)	Antibody 17b light chain
164	1ztx	1ztxEH	1ztx_E	1ztx_H	Envelope protein	Heavy Chain of E16 Antibody
165	1ztx	1ztxEL	1ztx_E	1ztx_L	Envelope protein	Light Chain of E16 Antibody
166	1zv5	1zv5AL	1zv5_A	1zv5_L	Immunoglobulin heavy chain antibody variable	Lysozyme C
167	1zvh	1zvhAL	1zvh_A	1zvh_L	Immunoglobulin heavy chain antibody variable	Lysozyme C
168	1zvy	1zvyAB	1zvy_A	1zvy_B	Immunoglobulin heavy chain antibody variable	Lysozyme C
169	2a6i	2a6iAP	2a6i_A	2a6i_P	Germline antibody 36-65 Fab light chain	Dodecapeptide: KLASIPTHTSPL
170	2a6i	2a6iBP	2a6i_B	2a6i_P	Germline antibody 36-65 Fab heavy chain	Dodecapeptide: KLASIPTHTSPL
171	2adf	2adfAH	2adf_A	2adf_H	Von Willebrand factor	82D6A3 IgG
172	2adf	2adfAL	2adf_A	2adf_L	Von Willebrand factor	82D6A3 IgG
173	2aep	2aepAH	2aep_A	2aep_H	neuraminidase	FAB heavy chain
174	2aep	2aepAL	2aep_A	2aep_L	neuraminidase	FAB light chain
175	2ap2	2ap2CQ	2ap2_C	2ap2_Q	PROTEIN (ANTIBODY (LIGHT CHAIN))	PROTEIN (P-GLYCOPROTEIN)
176	2ap2	2ap2DQ	2ap2_D	2ap2_Q	PROTEIN (ANTIBODY (HEAVY CHAIN))	PROTEIN (P-GLYCOPROTEIN)
177	2arj	2arjHQ	2arj_H	2arj_Q	YTS 105.18 antigen binding region Heavy chain	T-cell surface glycoprotein CD8 alpha chain
178	2arj	2arjLQ	2arj_L	2arj_Q	YTS 105.18 antigen binding region Light chain	T-cell surface glycoprotein CD8 alpha chain
179	2b1a	2b1aHP	2b1a_H	2b1a_P	Fab 2219, heavy chain	UG1033 peptide of Exterior membrane glycoprot
180	2b1a	2b1aLP	2b1a_L	2b1a_P	Fab 2219, light chain	UG1033 peptide of Exterior membrane glycoprot
181	2b1h	2b1hHP	2b1h_H	2b1h_P	Fab 2219, heavy chain	UG29 peptide of Exterior membrane glycoprotei
182	2b1h	2b1hLP	2b1h_L	2b1h_P	Fab 2219, light chain	UG29 peptide of Exterior membrane glycoprotei
183	2b2x	2b2xAH	2b2x_A	2b2x_H	Integrin alpha-1	Antibody AQC2 Fab
184	2b2x	2b2xAL	2b2x_A	2b2x_L	Integrin alpha-1	Antibody AQC2 Fab
185	2bdn	2bdnAH	2bdn_A	2bdn_H	Small inducible cytokine A2	Antibody heavy chain 11K2
186	2bdn	2bdnAL	2bdn_A	2bdn_L	Small inducible cytokine A2	Antibody light chain 11K2
187	2brr	2brrHP	2brr_H	2brr_P	MN20B9.34 ANTI-P1.4 ANTIBODY, FAB HEAVY CHAIN	CLASS 1 OUTER MEMBRANE PROTEIN VARIABLE REGIO
188	2brr	2brrLP	2brr_L	2brr_P	MN20B9.34 ANTI-P1.4 ANTIBODY, FAB LIGHT CHAIN	CLASS 1 OUTER MEMBRANE PROTEIN VARIABLE REGIO
189	2bse	2bseCF	2bse_C	2bse_F	RECEPTOR BINDING PROTEIN	LLAMA IMMUNOGLOBULIN
190	2ck0	2ck0HP	2ck0_H	2ck0_P	PROTEIN (IMMUNOGLOBULIN; HEAVY CHAIN)	PROTEIN (11-MER; CYCLIC PEPTIDE)
191	2ck0	2ck0LP	2ck0_L	2ck0_P	PROTEIN (IMMUNOGLOBULIN; LIGHT CHAIN)	PROTEIN (11-MER; CYCLIC PEPTIDE)
192	2dd8	2dd8HS	2dd8_H	2dd8_S	IGG Heavy Chain	Spike glycoprotein

193	2dd8	2dd8LS	2dd8_L	2dd8_S	IGG Light Chain	Spike glycoprotein
194	2dqc	2dqcHY	2dqc_H	2dqc_Y	Ig VH,anti-lysozyme	Lysozyme C
195	2dwd	2dwdBC	2dwd_B	2dwd_C	ANTIBODY FAB LIGHT CHAIN	Voltage-gated potassium channel
196	2f58	2f58HP	2f58_H	2f58_P	PROTEIN (IGG1 FAB 58.2 ANTIBODY (HEAVY CHAIIN	PROTEIN (HIV-1 GP120)
197	2fd6	2fd6HU	2fd6_H	2fd6_U	H chain of Fab of ATN-615 anti-uPAR antibody	Urokinase plasminogen activator surface recep
198	2fd6	2fd6LU	2fd6_L	2fd6_U	L chain of Fab of ATN-615 anti-uPAR antibody	Urokinase plasminogen activator surface recep
199	2fjg	2fjgAV	2fjg_A	2fjg_V	Fab light chain	Vascular endothelial growth factor A
200	2fjg	2fjgAW	2fjg_A	2fjg_W	Fab light chain	Vascular endothelial growth factor A
201	2fjg	2fjgBV	2fjg_B	2fjg_V	Fab heavy chain	Vascular endothelial growth factor A
202	2fjg	2fjgBW	2fjg_B	2fjg_W	Fab heavy chain	Vascular endothelial growth factor A
203	2ghw	2ghwAB	2ghw_A	2ghw_B	Spike glycoprotein	Anti-sars scFv antibody, 80R
204	2hmi	2hmiBC	2hmi_B	2hmi_C	HISUBUNIT OF V-1 REVERSE TRANSCRIPTASE	FAB FRAGMENT OF MONOCLONAL ANTIBODY 28
205	2hmi	2hmiBD	2hmi_B	2hmi_D	HISUBUNIT OF V-1 REVERSE TRANSCRIPTASE	FAB FRAGMENT OF MONOCLONAL ANTIBODY 28
206	2hrp	2hrpHP	2hrp_H	2hrp_P	MONOCLONAL ANTIBODY F11.2.32	HIV-1 PROTEASE PEPTIDE
207	2hrp	2hrpLP	2hrp_L	2hrp_P	MONOCLONAL ANTIBODY F11.2.32	HIV-1 PROTEASE PEPTIDE
208	2iff	2iffLY	2iff_L	2iff_Y	IGG1 HYHEL-5 FAB (LIGHT CHAIN)	HEN EGG WHITE LYSOZYME
209	2igf	2igfHP	2igf_H	2igf_P	IGG1-KAPPA B13I2 FAB (HEAVY CHAIN)	PEPTIDE (RESIDUES 69-87 OF MYOHEMERYTHRIN)
210	2igf	2igfLP	2igf_L	2igf_P	IGG1-KAPPA B13I2 FAB (LIGHT CHAIN)	PEPTIDE (RESIDUES 69-87 OF MYOHEMERYTHRIN)
211	2iwg	2iwgAB	2iwg_A	2iwg_B	IG GAMMA-1 CHAIN C	52 KDA RO PROTEIN
212	2j4w	2j4wDH	2j4w_D	2j4w_H	APICAL MEMBRANE ANTIGEN 1	FAB FRAGMENT OF MONOCLONAL ANTIBODY F8.12.19
213	2j4w	2j4wDL	2j4w_D	2j4w_L	APICAL MEMBRANE ANTIGEN 1	FAB FRAGMENT OF MONOCLONAL ANTIBODY F8.12.19
214	2j5l	2j5lAB	2j5l_A	2j5l_B	APICAL MEMBRANE ANTIGEN 1	FAB FRAGMENT OF MONOCLONAL ANTIBODY F8.12.19
215	2j5l	2j5lAC	2j5l_A	2j5l_C	APICAL MEMBRANE ANTIGEN 1	FAB FRAGMENT OF MONOCLONAL ANTIBODY F8.12.19
216	2jel	2jelHP	2jel_H	2jel_P	JEL42 FAB FRAGMENT	HISTIDINE-CONTAINING PROTEIN
217	2jel	2jelLP	2jel_L	2jel_P	JEL42 FAB FRAGMENT	HISTIDINE-CONTAINING PROTEIN
218	2ny7	2ny7GH	2ny7_G	2ny7_H	ENVELOPE GLYCOPROTEIN GP120	ANTIBODY b12, HEAVY CHAIN
219	2osl	2oslHP	2osl_H	2osl_P	Heavy chain of the Rituximab Fab fragment	B-lymphocyte antigen CD20
220	2osl	2oslLP	2osl_L	2osl_P	Light chain of the Rituximab Fab fragment	B-lymphocyte antigen CD20
221	2p4a	2p4aAB	2p4a_A	2p4a_B	Ribonuclease pancreatic	ANTIBODY CAB-RN05
222	2r56	2r56BI	2r56_B	2r56_I	Beta-lactoglobulin	IgE Fab Fragment, heavy chain
223	2r56	2r56BM	2r56_B	2r56_M	Beta-lactoglobulin	IgE Fab Fragment, light chain
224	2vdk	2vdkAH	2vdk_A	2vdk_H	INTEGRIN ALPHA-IIB	MONOCLONAL ANTIBODY 10E5 HEAVY CHAIN
225	2vdk	2vdkAL	2vdk_A	2vdk_L	INTEGRIN ALPHA-IIB	MONOCLONAL ANTIBODY 10E5 LIGHT CHAIN
226	2vol	2volAB	2vol_A	2vol_B	MURINE IGG FC	52 KDA RO PROTEIN
227	2vxq	2vxqAH	2vxq_A	2vxq_H	POLLEN ALLERGEN PHL P 2	FAB
228	2vxq	2vxqAL	2vxq_A	2vxq_L	POLLEN ALLERGEN PHL P 2	FAB
229	2vxs	2vxsCJ	2vxs_C	2vxs_J	INTERLEUKIN-17A	FAB FRAGMENT
230	2vxs	2vxsCO	2vxs_C	2vxs_O	INTERLEUKIN-17A	FAB FRAGMENT
231	2vxt	2vxtHI	2vxt_H	2vxt_I	MURINE IGG 125-2H	INTERLEUKIN-18

232	2vxt	2vxtIL	2vxt_I	2vxt_L	INTERLEUKIN-18	MURINE IGG 125-2H
233	2vyr	2vyrAE	2vyr_A	2vyr_E	MDM4 PROTEIN	HUMAN SINGLE DOMAIN ANTIBODY
234	2w9e	2w9eAH	2w9e_A	2w9e_H	MAJOR PRION PROTEIN	ICSM 18-ANTI-PRP THERAPEUTIC FAB HEAVY CHAIN
235	2w9e	2w9eAL	2w9e_A	2w9e_L	MAJOR PRION PROTEIN	ICSM 18-ANTI-PRP THERAPEUTIC FAB LIGHT CHAIN
236	2wuc	2wucAH	2wuc_A	2wuc_H	HEPATOCYTE GROWTH FACTOR ACTIVATOR LONG CHAIN	FAB FRAGMENT FAB40.DELTATRP HEAVY CHAIN
237	2wuc	2wucAL	2wuc_A	2wuc_L	HEPATOCYTE GROWTH FACTOR ACTIVATOR LONG CHAIN	FAB FRAGMENT FAB40.DELTATRP LIGHT CHAIN
238	2xqb	2xqbAH	2xqb_A	2xqb_H	INTERLEUKIN 15	ANTI-IL-15 ANTIBODY
239	2xqb	2xqbAL	2xqb_A	2xqb_L	INTERLEUKIN 15	ANTI-IL-15 ANTIBODY
240	2xqy	2xqyAG	2xqy_A	2xqy_G	ENVELOPE GLYCOPROTEIN H	A13-D6.3 MONOCLONAL ANTIBODY
241	2xqy	2xqyAL	2xqy_A	2xqy_L	ENVELOPE GLYCOPROTEIN H	A13-D6.3 MONOCLONAL ANTIBODY
242	2xra	2xraAH	2xra_A	2xra_H	TRANSMEMBRANE PROTEIN GP41	HK20, HUMAN MONOCLONAL ANTIBODY HEAVY CHAIN
243	2xra	2xraAL	2xra_A	2xra_L	TRANSMEMBRANE PROTEIN GP41	HK20, HUMAN MONOCLONAL ANTIBODY LIGHT CHAIN
244	2xtj	2xtjAB	2xtj_A	2xtj_B	PROPROTEIN CONVERTASE SUBTILISIN/KEXIN TYPE 9	FAB FROM A HUMAN MONOCLONAL ANTIBODY, 1D05
245	2xtj	2xtjAD	2xtj_A	2xtj_D	PROPROTEIN CONVERTASE SUBTILISIN/KEXIN TYPE 9	FAB FROM A HUMAN MONOCLONAL ANTIBODY, 1D05
246	2xwt	2xwtAC	2xwt_A	2xwt_C	THYROID BLOCKING HUMAN AUTOANTIBODY K1-70 HEA	THYROTROPIN RECEPTOR
247	2xwt	2xwtBC	2xwt_B	2xwt_C	THYROID BLOCKING HUMAN AUTOANTIBODY K1-70 LIG	THYROTROPIN RECEPTOR
248	2xzq	2xzqHP	2xzq_H	2xzq_P	ANTI-NP MURINE GERMLINE MONOCLONAL ANTIBODY B	PHAGE DISPLAY DERIVED ANTIGEN
249	2xzq	2xzqLP	2xzq_L	2xzq_P	ANTI-NP MURINE GERMLINE MONOCLONAL ANTIBODY B	PHAGE DISPLAY DERIVED ANTIGEN
250	2y06	2y06HP	2y06_H	2y06_P	ANTI-NP MURINE GERMLINE MONOCLONAL ANTIBODY B	PHAGE DISPLAY DERIVED ANTIGEN
251	2y06	2y06LP	2y06_L	2y06_P	ANTI-NP MURINE GERMLINE MONOCLONAL ANTIBODY B	PHAGE DISPLAY DERIVED ANTIGEN
252	2y07	2y07HP	2y07_H	2y07_P	ANTI-NP MURINE GERMLINE MONOCLONAL ANTIBODY B	PHAGE DISPLAY DERIVED ANTIGEN
253	2y07	2y07LP	2y07_L	2y07_P	ANTI-NP MURINE GERMLINE MONOCLONAL ANTIBODY B	PHAGE DISPLAY DERIVED ANTIGEN
254	2y36	2y36HP	2y36_H	2y36_P	ANTI-NP MURINE GERMLINE MONOCLONAL ANTIBODY B	DODECAPEPTIDE (DLWTTAIPTIPS)
255	2y36	2y36LP	2y36_L	2y36_P	ANTI-NP MURINE GERMLINE MONOCLONAL ANTIBODY B	DODECAPEPTIDE (DLWTTAIPTIPS)
256	2y6s	2y6sCP	2y6s_C	2y6s_P	LIGHT CHAIN	ENVELOPE GLYCOPROTEIN
257	2y6s	2y6sDP	2y6s_D	2y6s_P	HEAVY CHAIN	ENVELOPE GLYCOPROTEIN
258	2yc1	2yc1AC	2yc1_A	2yc1_C	SINGLE CHAIN ANTIBODY FRAGMENT 9004G	BETA-MAMMAL TOXIN CN2
259	2yc1	2yc1BC	2yc1_B	2yc1_C	SINGLE CHAIN ANTIBODY FRAGMENT 9004G	BETA-MAMMAL TOXIN CN2
260	2znw	2znwAY	2znw_A	2znw_Y	ScFv10	Lysozyme C
261	3bae	3baeAH	3bae_A	3bae_H	Amyloid Beta Peptide	WO2 IgG2a Fab fragment Heavy Chain
262	3bae	3baeAL	3bae_A	3bae_L	Amyloid Beta Peptide	WO2 IgG2a Fab fragment Light Chain Kappa
263	3be1	3be1AL	3be1_A	3be1_L	Receptor tyrosine-protein kinase erbB-2	Fab Fragment-Light Chain
264	3bgf	3bgfAB	3bgf_A	3bgf_B	Spike protein S1	F26G19 Fab
265	3bgf	3bgfAC	3bgf_A	3bgf_C	Spike protein S1	F26G19 Fab
266	3bn9	3bn9BC	3bn9_B	3bn9_C	Membrane-type serine protease 1	E2 Fab Light Chain
267	3bn9	3bn9BD	3bn9_B	3bn9_D	Membrane-type serine protease 1	E2 Fab Heavy Chain
268	3c2a	3c2aHP	3c2a_H	3c2a_P	Fab 447-52D, heavy chain	Envelope glycoprotein
269	3cvh	3cvhAH	3cvh_A	3cvh_H	H-2 class I histocompatibility antigen, K-B a	25-D1.16 heavy chain
270	3cvh	3cvhAL	3cvh_A	3cvh_L	H-2 class I histocompatibility antigen, K-B a	25-D1.16 light chain

271	3cxd	3cxdHP	3cxd_H	3cxd_P	Fab fragment of anti-osteopontin antibody 23C	A peptide from osteopontin
272	3cxd	3cxdLP	3cxd_L	3cxd_P	Fab fragment of anti-osteopontin antibody 23C	A peptide from osteopontin
273	3d0l	3d0lAC	3d0l_A	3d0l_C	2F5 heavy chain	Hyb3k peptide construct
274	3d85	3d85AC	3d85_A	3d85_C	FAB of antibody 7G10, light chain	Interleukin-23 subunit p19
275	3d85	3d85BC	3d85_B	3d85_C	FAB of antibody 7G10, heavy chain	Interleukin-23 subunit p19
276	3dsf	3dsfLP	3dsf_L	3dsf_P	Fab fragment of anti-osteopontin antibody 23C	A peptide from osteopontin
277	3dus	3dusAB	3dus_A	3dus_B	antibody Fv fragment SAG506-01	Ig-like protein
278	3dvg	3dvgAX	3dvg_A	3dvg_X	Human IgG1 fab fragment light chain	Ubiquitin D77
279	3dvg	3dvgAY	3dvg_A	3dvg_Y	Human IgG1 fab fragment light chain	Ubiquitin
280	3dvg	3dvgBX	3dvg_B	3dvg_X	Human IgG1 fab fragment heavy chain	Ubiquitin D77
281	3dvg	3dvgBY	3dvg_B	3dvg_Y	Human IgG1 fab fragment heavy chain	Ubiquitin
282	3e8u	3e8uHP	3e8u_H	3e8u_P	Fab 106.3 heavy chain	BNP peptide epitope
283	3e8u	3e8uLP	3e8u_L	3e8u_P	Fab 106.3 light chain	BNP peptide epitope
284	3eoa	3eoaHI	3eoa_H	3eoa_I	Efalizumab Fab fragment, heavy chain	Integrin alpha-L
285	3eoa	3eoaIL	3eoa_I	3eoa_L	Integrin alpha-L	Efalizumab Fab fragment, light chain
286	3ffd	3ffdAP	3ffd_A	3ffd_P	Monoclonal antibody, heavy chain, Fab fragmen	Parathyroid hormone-related protein
287	3ffd	3ffdBP	3ffd_B	3ffd_P	Monoclonal antibody, light chain, Fab fragmen	Parathyroid hormone-related protein
288	3g04	3g04AC	3g04_A	3g04_C	HUMAN THYROID STIMULATING AUTOANTIBODY M22 LI	Thyrotropin receptor
289	3g04	3g04BC	3g04_B	3g04_C	HUMAN THYROID STIMULATING AUTOANTIBODY M22 HE	Thyrotropin receptor
290	3g5v	3g5vAC	3g5v_A	3g5v_C	806 light chain	Epidermal Growth Factor Receptor peptide
291	3g5v	3g5vBC	3g5v_B	3g5v_C	808 heavy chain	Epidermal Growth Factor Receptor peptide
292	3gbm	3gbmAH	3gbm_A	3gbm_H	Hemagglutinin	Antibody (Fab)
293	3gbm	3gbmBH	3gbm_B	3gbm_H	Hemagglutinin	Antibody (Fab)
294	3gbn	3gbnAH	3gbn_A	3gbn_H	Hemagglutinin	Fab Heavy Chain
295	3ghb	3ghbHP	3ghb_H	3ghb_P	Fab 447-52D, heavy chain	Envelope glycoprotein
296	3ghe	3gheHP	3ghe_H	3ghe_P	Fab 537-10D, heavy chain	Envelope glycoprotein
297	3ghe	3gheLP	3ghe_L	3ghe_P	Fab 537-10D, light chain	Envelope glycoprotein
298	3gi8	3gi8CH	3gi8_C	3gi8_H	Uncharacterized protein MJ0609	7F11 Anti-ApcT Monoclonal Fab Heavy Chain
299	3gi8	3gi8CL	3gi8_C	3gi8_L	Uncharacterized protein MJ0609	7F11 Anti-ApcT Monoclonal Fab Light Chain
300	3gjf	3gjfAH	3gjf_A	3gjf_H	HLA class I histocompatibility antigen, A-2 a	Antibody heavy chain
301	3go1	3go1HP	3go1_H	3go1_P	Fab 268-D, heavy chain	Envelope glycoprotein gp160
302	3go1	3go1LP	3go1_L	3go1_P	Fab 268-D, light chain	Envelope glycoprotein gp160
303	3h0t	3h0tAC	3h0t_A	3h0t_C	Fab fragment, Light chain	Hepcidin
304	3h0t	3h0tBC	3h0t_B	3h0t_C	Fab fragment, Heavy chain	Hepcidin
305	3h3b	3h3bAC	3h3b_A	3h3b_C	Receptor tyrosine-protein kinase erbB-2	Anti-ErbB2 antibody chA21
306	3h3p	3h3pHS	3h3p_H	3h3p_S	Fv 4E10 heavy chain	4E10_S0_1TJLC_004_N
307	3hae	3haeAH	3hae_A	3hae_H	HLA class I histocompatibility antigen, A-2 a	Antibody heavy chain
308	3hae	3haeAL	3hae_A	3hae_L	HLA class I histocompatibility antigen, A-2 a	Antibody light chain
309	3hi6	3hi6BX	3hi6_B	3hi6_X	Integrin alpha-L	Heavy chain of Fab fragment of AL-57 against

310	3hi6	3hi6BY	3hi6_B	3hi6_Y	Integrin alpha-L	Light chain of Fab fragment of AL-57 against
311	3hr5	3hr5JV	3hr5_J	3hr5_V	Fab h47H4 heavy chain	M1prime-derived peptide
312	3hr5	3hr5QV	3hr5_Q	3hr5_V	Fab h47H4 light chain	M1prime-derived peptide
313	3idx	3idxGH	3idx_G	3idx_H	HIV-1 HxBc2 gp120 core	Fab b13 heavy chain
314	3ifn	3ifnHP	3ifn_H	3ifn_P	12A11 FAB antibody heavy chain	Amyloid beta A4 protein
315	3iu3	3iu3AK	3iu3_A	3iu3_K	Heavy chain of Fab fragment of Basiliximab	Interleukin-2 receptor alpha chain
316	3iu3	3iu3BK	3iu3_B	3iu3_K	Light chain of Fab fragment of Basiliximab	Interleukin-2 receptor alpha chain
317	3jwd	3jwdAH	3jwd_A	3jwd_H	HIV-1 GP120 ENVELOPE GLYCOPROTEIN	FAB 48D HEAVY CHAIN
318	3jwd	3jwdAL	3jwd_A	3jwd_L	HIV-1 GP120 ENVELOPE GLYCOPROTEIN	FAB 48D LIGHT CHAIN
319	3k7u	3k7uAC	3k7u_A	3k7u_C	Antibody	MP18 RNA editing complex protein
320	3k80	3k80AC	3k80_A	3k80_C	Antibody	MP18 RNA editing complex protein
321	3kr3	3kr3DH	3kr3_D	3kr3_H	Insulin-like growth factor II	Antibody-Fab (heavy chain)
322	3kr3	3kr3DL	3kr3_D	3kr3_L	Insulin-like growth factor II	Antibody-Fab (light chain)
323	3l5w	3l5wHI	3l5w_H	3l5w_I	C836 HEAVY CHAIN	Interleukin-13
324	3l5w	3l5wIL	3l5w_I	3l5w_L	Interleukin-13	C836 LIGHT CHAIN
325	3l95	3l95AX	3l95_A	3l95_X	anti-NRR1 fab fragment light chain	Neurogenic locus notch homolog protein 1
326	3l95	3l95BX	3l95_B	3l95_X	anti-NRR1 fab fragment heavy chain	Neurogenic locus notch homolog protein 1
327	3ldb	3ldbAB	3ldb_A	3ldb_B	Bifunctional arginine demethylase and lysyl-h	Antibody Fab fragment light chain
328	3ldb	3ldbAC	3ldb_A	3ldb_C	Bifunctional arginine demethylase and lysyl-h	Antibody Fab fragment heavy chain
329	3lev	3levAH	3lev_A	3lev_H	RNA polymerase sigma factor	2F5 ANTIBODY HEAVY CHAIN
330	3lh2	3lh2KS	3lh2_K	3lh2_S	Fv 4E10 heavy chain	4E10_1VI7A_S0_002_N (T88)
331	3lh2	3lh2OS	3lh2_O	3lh2_S	Fv 4E10 light chain	4E10_1VI7A_S0_002_N (T88)
332	3lhp	3lhpHS	3lhp_H	3lhp_S	Fv 4E10 heavy chain	4E10_D0_1ISEA_004_N (T93)
333	3lhp	3lhpLS	3lhp_L	3lhp_S	Fv 4E10 light chain	4E10_D0_1ISEA_004_N (T93)
334	3liz	3lizAH	3liz_A	3liz_H	Aspartic protease Bla g 2	4C3 monoclonal antibody Heavy Chain
335	3liz	3lizAL	3liz_A	3liz_L	Aspartic protease Bla g 2	4C3 monoclonal antibody Light Chain
336	3lzf	3lzfAH	3lzf_A	3lzf_H	Hemagglutinin, HA1 Subunit	2D1 Fab, Heavy Chain
337	3lzf	3lzfAL	3lzf_A	3lzf_L	Hemagglutinin, HA1 Subunit	2D1 Fab, Light Chain
338	3mlr	3mlrHP	3mlr_H	3mlr_P	Human monoclonal anti-HIV-1 gp120 V3 antibody	HIV-1 gp120 third variable region (V3) crown
339	3mlr	3mlrLP	3mlr_L	3mlr_P	Human monoclonal anti-HIV-1 gp120 V3 antibody	HIV-1 gp120 third variable region (V3) crown
340	3mls	3mlsHP	3mls_H	3mls_P	Human monoclonal anti-HIV-1 gp120 V3 antibody	Rationally designed V3 mimotope
341	3mlt	3mltLP	3mlt_L	3mlt_P	Human monoclonal anti-HIV-1 gp120 V3 antibody	HIV-1 gp120 third variable region (V3) crown
342	3mlu	3mluLP	3mlu_L	3mlu_P	Human monoclonal anti-HIV-1 gp120 V3 antibody	HIV-1 gp120 third variable region (V3) crown
343	3mlv	3mlvLP	3mlv_L	3mlv_P	Human monoclonal anti-HIV-1 gp120 V3 antibody	HIV-1 gp120 third variable region (V3) crown
344	3mlw	3mlwHP	3mlw_H	3mlw_P	Human monoclonal anti-HIV-1 gp120 V3 antibody	HIV-1 gp120 third variable region (V3) crown
345	3mlx	3mlxHP	3mlx_H	3mlx_P	Human monoclonal anti-HIV-1 gp120 V3 antibody	HIV-1 gp120 third variable region (V3) crown
346	3mlx	3mlxLP	3mlx_L	3mlx_P	Human monoclonal anti-HIV-1 gp120 V3 antibody	HIV-1 gp120 third variable region (V3) crown
347	3mly	3mlyHP	3mly_H	3mly_P	Human monoclonal anti-HIV-1 gp120 V3 antibody	HIV-1 gp120 third variable region (V3) crown
348	3mly	3mlyLP	3mly_L	3mly_P	Human monoclonal anti-HIV-1 gp120 V3 antibody	HIV-1 gp120 third variable region (V3) crown

349	3mlz	3mlzHP	3mlz_H	3mlz_P	Human monoclonal anti-HIV-1 gp120 V3 antibody	HIV-1 gp120 third variable region (V3) crown
350	3mlz	3mlzLP	3mlz_L	3mlz_P	Human monoclonal anti-HIV-1 gp120 V3 antibody	HIV-1 gp120 third variable region (V3) crown
351	3mnw	3mnwAP	3mnw_A	3mnw_P	ANTI-HIV-1 ANTIBODY 13H11 LIGHT CHAIN	Gp41
352	3mnw	3mnwBP	3mnw_B	3mnw_P	ANTI-HIV-1 ANTIBODY 13H11 HEAVY CHAIN	Gp41
353	3mxw	3mxwAH	3mxw_A	3mxw_H	Sonic hedgehog protein	5E1 heavy chain
354	3mxw	3mxwAL	3mxw_A	3mxw_L	Sonic hedgehog protein	5E1 light chain
355	3ngb	3ngbGH	3ngb_G	3ngb_H	Envelope glycoprotein gp160	Antigen binding fragment of heavy chain: Anti
356	3ngb	3ngbGL	3ngb_G	3ngb_L	Envelope glycoprotein gp160	Antigen binding fragment of light chain: Anti
357	3nh7	3nh7AH	3nh7_A	3nh7_H	Bone morphogenetic protein receptor type-1A	Antibody fragment Fab AbD1556, heavy chain
358	3nh7	3nh7AL	3nh7_A	3nh7_L	Bone morphogenetic protein receptor type-1A	Antibody fragment Fab AbD1556, light chain
359	3nps	3npsAB	3nps_A	3nps_B	Suppressor of tumorigenicity 14 protein	S4 FAB HEAVY CHAIN
360	3nps	3npsAC	3nps_A	3nps_C	Suppressor of tumorigenicity 14 protein	S4 FAB LIGHT CHAIN
361	3o2d	3o2dAH	3o2d_A	3o2d_H	T-cell surface glycoprotein CD4	Ibalizumab heavy chain
362	3o2d	3o2dAL	3o2d_A	3o2d_L	T-cell surface glycoprotein CD4	Ibalizumab light chain
363	3o41	3o41HP	3o41_H	3o41_P	Mouse monoclonal antibody 101F Fab heavy chai	Fusion glycoprotein F1
364	3o45	3o45LP	3o45_L	3o45_P	Mouse monoclonal antibody 101F 101F Fab light	Fusion glycoprotein F1
365	3pgf	3pgfAH	3pgf_A	3pgf_H	Maltose-binding periplasmic protein	SAB Heavy Chain
366	3pgf	3pgfAL	3pgf_A	3pgf_L	Maltose-binding periplasmic protein	SAB Light Chain
367	3pp4	3pp4HP	3pp4_H	3pp4_P	GA101 Fab heavy chain	B-lymphocyte antigen CD20
368	3pp4	3pp4LP	3pp4_L	3pp4_P	GA101 Fab light chain	B-lymphocyte antigen CD20
369	3qwo	3qwoHP	3qwo_H	3qwo_P	Motavizumab heavy chain	Motavizumab epitope scaffold
370	3qwo	3qwoLP	3qwo_L	3qwo_P	Motavizumab light chain	Mmotavizumab epitope scaffold
371	3r1g	3r1gBH	3r1g_B	3r1g_H	Beta-secretase 1	FAB of YW412.8.31 antibody heavy chain
372	3r1g	3r1gBL	3r1g_B	3r1g_L	Beta-secretase 1	FAB of YW412.8.31 antibody light chain
373	3rvv	3rvvAC	3rvv_A	3rvv_C	Peptidase 1	4C1 - light chain
374	3rvv	3rvvAD	3rvv_A	3rvv_D	Peptidase 1	4C1 - heavy chain
375	3rvw	3rvwAC	3rvw_A	3rvw_C	Peptidase 1	4C1 - light chain
376	3rvw	3rvwAD	3rvw_A	3rvw_D	Peptidase 1	4C1 - heavy chain
377	3s35	3s35HX	3s35_H	3s35_X	6.64 Fab heavy chain	Vascular endothelial growth factor receptor 2
378	3s35	3s35LX	3s35_L	3s35_X	6.64 Fab light chain	Vascular endothelial growth factor receptor 2
379	3s37	3s37HX	3s37_H	3s37_X	1121B Fab heavy chain	Vascular endothelial growth factor receptor 2
380	3s37	3s37LX	3s37_L	3s37_X	1121B Fab light chain	Vascular endothelial growth factor receptor 2
381	3sdy	3sdyAH	3sdy_A	3sdy_H	Hemagglutinin HA1 chain	Antibody CR8020, Heavy Chain
382	3sdy	3sdyBH	3sdy_B	3sdy_H	Hemagglutinin HA2 chain	Antibody CR8020, Heavy Chain
383	3sdy	3sdyBL	3sdy_B	3sdy_L	Hemagglutinin HA2 chain	Antibody CR8020, Light Chain
384	3se8	3se8GH	3se8_G	3se8_H	HIV-1 Clade AE strain 93TH057 gp120	Heavy chain of antibody VRC03
385	3se8	3se8GL	3se8_G	3se8_L	HIV-1 Clade AE strain 93TH057 gp120	Light chain of antibody VRC03
386	3skj	3skjEH	3skj_E	3skj_H	Ephrin type-A receptor 2	Antibody, heavy chain
387	3skj	3skjEL	3skj_E	3skj_L	Ephrin type-A receptor 2	Antibody, light chain

388	3sob	3sobBH	3sob_B	3sob_H	Low-density lipoprotein receptor-related prot	Antibody heavy chain
389	3sob	3sobBL	3sob_B	3sob_L	Low-density lipoprotein receptor-related prot	Antibody light chain
390	3t2n	3t2nAH	3t2n_A	3t2n_H	Serine protease hepsin	Antibody, Fab fragment, Heavy Chain
391	3t2n	3t2nAL	3t2n_A	3t2n_L	Serine protease hepsin	Antibody, Fab fragment, Light Chain
392	3u0t	3u0tCE	3u0t_C	3u0t_E	ponezumab LC Fab	Amyloid beta A4 protein
393	3u0t	3u0tDE	3u0t_D	3u0t_E	ponezumab HC Fab	Amyloid beta A4 protein
394	3u30	3u30AB	3u30_A	3u30_B	Linear di-ubiquitin	Light chain Fab
395	3u30	3u30AC	3u30_A	3u30_C	Linear di-ubiquitin	Heavy chain Fab
396	3ztn	3ztnAH	3ztn_A	3ztn_H	HAEMAGGLUTININ	FI6V3 ANTIBODY LIGHT CHAIN
397	3ztn	3ztnBH	3ztn_B	3ztn_H	HAEMAGGLUTININ	FI6V3 ANTIBODY LIGHT CHAIN
398	3ztn	3ztnBL	3ztn_B	3ztn_L	HAEMAGGLUTININ	FI6V3 ANTIBODY LIGHT CHAIN
