# Supplementary material for: Genome-wide structural modelling of TCR-pMHC interactions
Source: BMC Genomics. 2013 Oct 16;14(Suppl 5):S5. doi: 10.1186/1471-2164-14-S5-S5 (PMC3852114; doi:10.1186/1471-2164-14-S5-S5)
Supplement: Additional file 5 — The 70 mutated residues with free energy changes in 4 Ag-Ab interfaces. The corresponding ΔΔG value indicates the change in free energy of binding upon mutation to alanine for each experimentally mutated residue derived from the ASEdb. [file 1471-2164-14-S5-S5-S5.pdf]

Table S3 - The 70 mutated residues with free energy changes in 4 Ag-Ab interfaces

Tag	PDB entry	Chain	Mutated amino acid	Position	ddG
1	1ahw	C	Tyr	156	4
2	1ahw	C	Thr	167	0
3	1ahw	C	Thr	197	1.3
4	1ahw	C	Val	198	-0.3
5	1dvf	D	Tyr	101	4.7
6	1dvf	B	Asp	54	4.3
7	1dvf	D	Gln	103	1.6
8	1dvf	D	Arg	105	4.1
9	1dvf	B	Trp	52	4.2
10	1dvf	B	Tyr	101	4
11	1dvf	D	Asn	55	1.9
12	1dvf	D	Ile	100	2.7
13	1dvf	B	Arg	99	1.9
14	1dvf	A	Trp	92	0.3
15	1dvf	C	Tyr	49	1.9
16	1dvf	B	Asn	56	1.2
17	1dvf	B	Asp	100	2.8
18	1dvf	D	His	33	1.9
19	1dvf	B	Asp	58	1.6
20	1dvf	B	Glu	98	4.2
21	1dvf	A	Tyr	32	2
22	1dvf	A	Tyr	49	1.7
23	1dvf	A	Tyr	50	0.7
24	1dvf	D	Asp	52	1.7
25	1dvf	B	Thr	30	0.9
26	1dvf	B	Tyr	32	1.8
27	1dvf	A	His	30	1.7
28	1dvf	D	Lys	30	1
29	1dvf	A	Ser	93	1.2
30	1vfb	C	Gln	121	2.9
31	1vfb	B	Tyr	101	4
32	1vfb	B	Asp	100	3.1
33	1vfb	C	Asn	19	0.3
34	1vfb	A	Trp	92	1.71
35	1vfb	C	Ser	24	0.8
36	1vfb	C	Arg	125	1.8
37	1vfb	B	Trp	52	1.23
38	1vfb	A	Tyr	32	1.3

39	1vfb	C	Asp	119	1
40	1vfb	C	Val	120	0.9
41	1vfb	C	Ile	124	1.2
42	1vfb	B	Asp	54	1.95
43	1vfb	A	Tyr	50	0.4
44	1vfb	A	Thr	53	-0.23
45	1vfb	A	Ser	93	0.11
46	1vfb	C	Asp	18	0.3
47	1vfb	C	Lys	116	0.7
48	1vfb	C	Thr	118	0.8
49	1vfb	B	Tyr	32	0.5
50	1vfb	B	Arg	99	0.47
51	1vfb	A	His	30	0.8
52	1vfb	A	Tyr	49	0.8
53	1vfb	C	Tyr	23	0.4
54	1vfb	C	Leu	129	0.2
55	1vfb	B	Thr	30	0.09
56	3hfm	Y	Tyr	20	4.2
57	3hfm	Y	Arg	21	0.85
58	3hfm	Y	Asp	101	0.94
59	3hfm	Y	Arg	73	-0.33
60	3hfm	Y	Leu	75	0.69
61	3hfm	Y	Lys	96	6.38
62	3hfm	Y	Ser	100	0.26
63	3hfm	H	Tyr	53	3.29
64	3hfm	H	Tyr	58	1.7
65	3hfm	Y	Asn	93	0.21
66	3hfm	Y	Lys	97	5.5
67	3hfm	Y	Trp	63	0.31
68	3hfm	Y	His	15	-0.44
69	3hfm	Y	Thr	89	0
70	3hfm	Y	Ile	98	0
