# Supplementary material for: Genome-wide structural modelling of TCR-pMHC interactions
Source: BMC Genomics. 2013 Oct 16;14(Suppl 5):S5. doi: 10.1186/1471-2164-14-S5-S5 (PMC3852114; doi:10.1186/1471-2164-14-S5-S5)
Supplement: Additional file 6 — The 17 TCR-peptide-HLA-A0201 complexes from the PDB. This table contains PDB entry, chains of TCR, peptide, and HLA-A0201. [file 1471-2164-14-S5-S5-S6.pdf]

Table S4 - The 17 TCR-peptide-HLA-A0201 complexes from the PDB

Tag	PDB entry	TCR chain	peptide chain	HLA-A0201 chain
1	1ao7	D, E	C	A
2	1bd2	D, E	C	A
3	1lp9	E, F	C	A
4	1oga	D,E	C	A
5	1qrn	D,E	C	A
6	1qse	D,E	C	A
7	1qsf	D,E	C	A
8	2bnq	D,E	C	A
9	2bnr	D,E	C	A
10	2f53	D,E	C	A
11	2f54	D,E	C	A
12	2p5e	D,E	C	A
13	2p5w	D,E	C	A
14	2pye	D,E	C	A
15	2vlj	D,E	C	A
16	2vlk	D,E	C	A
17	3gsn	A,B	P	H
