# Supplementary material for: Genome-wide structural modelling of TCR-pMHC interactions
Source: BMC Genomics. 2013 Oct 16;14(Suppl 5):S5. doi: 10.1186/1471-2164-14-S5-S5 (PMC3852114; doi:10.1186/1471-2164-14-S5-S5)
Supplement: Additional file 7 — The homologous peptide antigens in 389 pathogens with positive and negative hits recorded in the IEDB. This table provides the precision, the number of predicted homologous peptide antigens, and the positive and negative hits recorded in the IEDB for 389 pathogens. [file 1471-2164-14-S5-S5-S7.pdf]

Table S5 - The homologous peptide antigens in 389 pathogens with positive and negative hits recorded in the IEDB

Organism	Taxonomy ID	Taxonomy group	No. of predicted peptide antigen	Positive hits	Negative hits	Precision
Vaccinia virus Copenhagen	10249	Viruses	639	66	35	0.653
Vaccinia virus WR	10254	Viruses	621	63	33	0.656
SARS coronavirus	227859	Viruses	114	12	0	1
Hepatitis C virus (isolate BEBE1)	356413	Viruses	19	8	0	1
Mycobacterium tuberculosis	1773	Bacteria	5678	7	3	0.7
Mycobacterium tuberculosis H37Ra	419947	Bacteria	5421	7	2	0.778
Mycobacterium tuberculosis F11	336982	Bacteria	5418	7	2	0.778
Mycobacterium bovis	1765	Bacteria	5334	6	2	0.75
Mycobacterium bovis BCG str. Pasteur 1173P2	410289	Bacteria	5287	6	2	0.75
Shigella flexneri	623	Bacteria	7916	5	4	0.556
Shigella flexneri 2002017	591020	Bacteria	7818	5	4	0.556
Shigella dysenteriae Sd197	300267	Bacteria	6442	5	3	0.625
Zaire ebolavirus - Zaire (1995)	128951	Viruses	41	5	1	0.833
Ebola virus - Mayinga, Zaire, 1976	128952	Viruses	40	5	1	0.833
Hepatitis C virus (isolate Japanese)	11116	Viruses	21	5	0	1
Hepatitis C virus (isolate BK)	11105	Viruses	20	5	0	1
Shigella sonnei Ss046	300269	Bacteria	7724	4	4	0.5
Shigella boydii CDC 3083-94	344609	Bacteria	7013	4	4	0.5
Hepatitis B virus	10407	Viruses	1189	4	1	0.8
Hepatitis C virus (isolate 1)	11104	Viruses	26	4	0	1
Hepatitis C virus (isolate Taiwan)	31645	Viruses	23	4	0	1
Hepatitis C virus (isolate Con1)	333284	Viruses	20	4	0	1
Escherichia coli O157:H7	83334	Bacteria	10194	3	4	0.429
Escherichia coli UTI89	364106	Bacteria	10150	3	4	0.429
Escherichia coli O6	217992	Bacteria	10143	3	4	0.429
Escherichia coli E24377A	331111	Bacteria	9411	3	4	0.429
Escherichia coli 536	362663	Bacteria	9254	3	4	0.429
Shigella boydii Sb227	300268	Bacteria	7099	3	4	0.429
Mycobacterium avium 104	243243	Bacteria	6494	3	1	0.75
Mycobacterium avium subsp. paratuberculosis	1770	Bacteria	6026	3	1	0.75
Mycobacterium ulcerans Agy99	362242	Bacteria	5375	3	2	0.6
Deerpox virus W-848-83	305674	Viruses	649	3	2	0.6
Human herpesvirus 5 strain AD169	10360	Viruses	539	3	0	1
Human papillomavirus type 16	333760	Viruses	76	3	0	1
Human herpesvirus 5 strain Towne	10363	Viruses	64	3	0	1
Measles virus strain Edmonston	11235	Viruses	39	3	0	1
Hantaan virus 76-118	11602	Viruses	37	3	0	1
Machupo virus	11628	Viruses	33	3	0	1

Guanarito virus	45219	Viruses	31	3	0	1
Hepatitis C virus isolate HC-J6	11113	Viruses	24	3	0	1
Hepatitis C virus (isolate Th580)	356421	Viruses	24	3	0	1
Lassa virus Josiah	11622	Viruses	23	3	1	0.75
Hepatitis C virus (isolate H)	11108	Viruses	21	3	0	1
Escherichia coli APEC O1	405955	Bacteria	10195	2	4	0.333
Salmonella enterica	28901	Bacteria	9710	2	2	0.5
Yersinia pseudotuberculosis	633	Bacteria	8878	2	1	0.667
Salmonella enterica subsp. arizonae serovar 62:z4,z23:-	41514	Bacteria	8846	2	2	0.5
Yersinia pestis	632	Bacteria	8691	2	1	0.667
Yersinia pestis Antiqua	360102	Bacteria	8168	2	1	0.667
Yersinia pestis Nepal516	377628	Bacteria	8115	2	1	0.667
Mycobacterium vanbaalenii PYR-1	350058	Bacteria	7949	2	1	0.667
Mycobacterium sp. KMS	189918	Bacteria	7623	2	1	0.667
Mycobacterium sp. JLS	164757	Bacteria	7610	2	1	0.667
Mycobacterium sp. MCS	164756	Bacteria	7356	2	1	0.667
Shigella flexneri 5 str. 8401	373384	Bacteria	7345	2	4	0.333
Yersinia pestis Angola	349746	Bacteria	7131	2	1	0.667
Coxiella burnetii Dugway 5J108-111	434922	Bacteria	4647	2	0	1
Francisella tularensis subsp. tularensis	119856	Bacteria	4124	2	0	1
Francisella tularensis subsp. tularensis FSC198	393115	Bacteria	4119	2	0	1
Francisella tularensis subsp. tularensis WY96-3418	418136	Bacteria	4112	2	0	1
Coxiella burnetii	777	Bacteria	4028	2	0	1
Coxiella burnetii RSA 331	360115	Bacteria	3942	2	0	1
Coxiella burnetii CbuG_Q212	434923	Bacteria	3915	2	0	1
Mycobacterium leprae	1769	Bacteria	2600	2	2	0.5
Influenza A virus (A/gray teal/Australia/2/1979(H4N4))	402464	Viruses	36	2	4	0.333
Marburg virus - Musoke, Kenya, 1980	33727	Viruses	34	2	2	0.5
Influenza A virus (A/Puerto Rico/8/1934(H1N1))	211044	Viruses	34	2	3	0.4
Ravn virus - Ravn, Kenya, 1987	378809	Viruses	34	2	1	0.667
Influenza A virus (A/Chile/1/1983(H1N1))	380985	Viruses	34	2	3	0.4
Influenza A virus (A/Wilson-Smith/1933(H1N1))	381518	Viruses	33	2	3	0.4
Influenza A virus (A/chicken/Scotland/1959(H5N1))	402527	Viruses	33	2	4	0.333
Lake Victoria marburgvirus - Popp	33728	Viruses	32	2	2	0.5
Influenza A virus (A/Memphis/1/1971(H3N2))	383586	Viruses	32	2	3	0.4
Influenza A virus (A/Goose/Guangdong/1/96(H5N1))	93838	Viruses	31	2	2	0.5
Influenza A virus (A/Hong Kong/156/97(H5N1))	130763	Viruses	31	2	3	0.4
Influenza A virus (A/duck/England/1/1956(H11N6))	383550	Viruses	31	2	4	0.333
Influenza A virus (A/Memphis/102/1972(H3N2))	385640	Viruses	31	2	3	0.4
Influenza A virus (A/duck/Hokkaido/8/1980(H3N8))	387207	Viruses	31	2	4	0.333

Influenza A virus (A/Udorn/307/1972(H3N2))	381517	Viruses	29	2	3	0.4
Sabia virus	45709	Viruses	28	2	0	1
Cupixi virus	208899	Viruses	26	2	0	1
HIV-1 M:B_ARV2/SF2	11685	Viruses	24	2	0	1
Hepatitis C virus (isolate JK049)	356417	Viruses	22	2	0	1
Lymphocytic choriomeningitis virus (strain Armstrong)	11624	Viruses	21	2	1	0.667
Human T-cell lymphotrophic virus type 1 (strain ATK)	11926	Viruses	21	2	0	1
Hepatitis C virus (isolate HCV-K3a/650)	356416	Viruses	21	2	0	1
Hepatitis C virus (isolate 6a33)	356391	Viruses	20	2	0	1
Hepatitis C virus (isolate NZL1)	356415	Viruses	19	2	0	1
Hepatitis C virus (isolate HC-G9)	356410	Viruses	16	2	0	1
Hepatitis B virus alpha1	10411	Viruses	12	2	1	0.667
Bacillus cereus E33L	288681	Bacteria	12393	1	2	0.333
Bacillus weihenstephanensis KBAB4	315730	Bacteria	12112	1	1	0.5
Bacillus anthracis	1392	Bacteria	12080	1	2	0.333
Bacillus cereus G9842	405531	Bacteria	11986	1	1	0.5
Bacillus cereus ATCC 10987	222523	Bacteria	11876	1	1	0.5
Bacillus anthracis str. CDC 684	568206	Bacteria	11811	1	2	0.333
Bacillus cereus 03BB102	572264	Bacteria	11638	1	2	0.333
Bacillus thuringiensis str. Al Hakam	412694	Bacteria	11086	1	2	0.333
Bacillus cereus ATCC 14579	226900	Bacteria	11052	1	1	0.5
Salmonella enterica subsp. enterica serovar Typhimurium	90371	Bacteria	9900	1	2	0.333
Vibrio parahaemolyticus	670	Bacteria	9805	1	1	0.5
Salmonella enterica subsp. enterica serovar Newport str. SL254	423368	Bacteria	9122	1	2	0.333
Yersinia pseudotuberculosis IP 31758	349747	Bacteria	8506	1	1	0.5
Escherichia coli HS	331112	Bacteria	8475	1	3	0.25
Bacillus cytotoxicus NVH 391-98	315749	Bacteria	8361	1	0	1
Yersinia enterocolitica subsp. enterocolitica 8081	393305	Bacteria	8206	1	1	0.5
Listeria monocytogenes	1639	Bacteria	6241	1	0	1
Staphylococcus aureus subsp. aureus NCTC 8325	93061	Bacteria	6078	1	0	1
Campylobacter jejuni	197	Bacteria	6024	1	0	1
Staphylococcus aureus subsp. aureus MRSA252	282458	Bacteria	5921	1	0	1
Staphylococcus aureus subsp. aureus COL	93062	Bacteria	5900	1	0	1
Staphylococcus aureus subsp. aureus N315	158879	Bacteria	5897	1	0	1
Listeria monocytogenes serotype 4b str. CLIP 80459	568819	Bacteria	5803	1	0	1
Listeria monocytogenes serotype 4b str. F2365	265669	Bacteria	5770	1	0	1
Campylobacter jejuni RM1221	195099	Bacteria	4537	1	0	1
Campylobacter jejuni subsp. jejuni 81-176	354242	Bacteria	4467	1	0	1
Haemophilus influenzae	727	Bacteria	4363	1	0	1

Campylobacter jejuni subsp. jejuni 81116	407148	Bacteria	4334	1	0	1
Francisella tularensis subsp. holarctica LVS	376619	Bacteria	4201	1	0	1
Francisella tularensis subsp. holarctica OSU18	393011	Bacteria	3837	1	0	1
Corynebacterium jeikeium K411	306537	Bacteria	2947	1	0	1
Rickettsia conorii	781	Bacteria	2839	1	0	1
Rickettsia massiliae MTU5	416276	Bacteria	2629	1	0	1
Rickettsia rickettsii str. 'Sheila Smith'	392021	Bacteria	2556	1	0	1
Rickettsia prowazekii	782	Bacteria	2458	1	0	1
Rickettsia typhi	785	Bacteria	2421	1	0	1
Simian immunodeficiency virus	11723	Viruses	1461	1	0	1
Human herpesvirus 4	10376	Viruses	289	1	1	0.5
Human herpesvirus 4 (strain B95-8)	10377	Viruses	235	1	1	0.5
Herpes simplex virus (type 1 / strain 17)	10299	Viruses	200	1	0	1
Reston ebolavirus - Reston	129003	Viruses	61	1	1	0.5
Canine distemper virus strain Onderstepoort	11233	Viruses	41	1	0	1
Human respiratory syncytial virus A2	11259	Viruses	37	1	0	1
Rinderpest virus (strain RBOK)	36409	Viruses	35	1	0	1
Puumala virus (STRAIN HALLNAS B1)	11605	Viruses	33	1	0	1
Human T-lymphotropic virus 2	11909	Viruses	32	1	0	1
Rabies virus AVO1	11293	Viruses	31	1	0	1
BK polyomavirus	10629	Viruses	30	1	0	1
Human immunodeficiency virus type 1 (ELI ISOLATE)	11689	Viruses	22	1	0	1
Hepatitis C virus (isolate Tr Kj)	357355	Viruses	21	1	0	1
Human immunodeficiency virus type 1 (RF/HAT ISOLATE)	11701	Viruses	20	1	0	1
Human immunodeficiency virus type 1 BH10	11678	Viruses	19	1	0	1
Human immunodeficiency virus type 1 (JRCSF ISOLATE)	11688	Viruses	19	1	0	1
HIV-1 M:B_MN	11696	Viruses	19	1	0	1
HIV-1 M:B_HXB2R	11706	Viruses	19	1	0	1
Human hepatitis A virus Hu/Australia/HM175/1976	12098	Viruses	19	1	0	1
Dengue virus 4 Thailand/0348/1991	408688	Viruses	18	1	0	1
Human T-cell lymphotrophic virus type 1 (Caribbean isolate)	11927	Viruses	17	1	0	1
Simian virus 40	10633	Viruses	16	1	0	1
Human immunodeficiency virus type 1 (BRU ISOLATE)	11686	Viruses	16	1	0	1
Dengue virus 3 Philippines/H87/1956	408870	Viruses	16	1	0	1
Human immunodeficiency virus type 1 (MAL ISOLATE)	11697	Viruses	13	1	0	1
Hepatitis B virus LSH/chimpanzee	10414	Viruses	11	1	1	0.5
Candidatus Solibacter usitatus Ellin6076	234267	Bacteria	16709	0	0	-
Nostoc punctiforme PCC 73102	63737	Bacteria	14542	0	0	-
Pseudomonas aeruginosa	287	Bacteria	13119	0	1	0
Burkholderia sp. 383	269483	Bacteria	13037	0	0	-

Burkholderia vietnamiensis G4	269482	Bacteria	11615	0	0	-
Bradyrhizobium sp. BTAi1	288000	Bacteria	11320	0	1	0
Burkholderia ambifaria AMMD	339670	Bacteria	11021	0	0	-
Clostridium beijerinckii NCIMB 8052	290402	Bacteria	10936	0	0	-
Burkholderia cenocepacia AU 1054	331271	Bacteria	10746	0	0	-
Desulfatibacillum alkenivorans AK-01	439235	Bacteria	10745	0	0	-
Burkholderia pseudomallei 1710b	320372	Bacteria	10563	0	0	-
Pseudomonas aeruginosa UCBPP-PA14	208963	Bacteria	10398	0	0	-
Cupriavidus metallidurans CH34	266264	Bacteria	10262	0	0	-
Burkholderia pseudomallei	28450	Bacteria	10222	0	0	-
Vibrio vulnificus YJ016	196600	Bacteria	10222	0	1	0
Burkholderia pseudomallei 668	320373	Bacteria	10138	0	0	-
Sinorhizobium meliloti	382	Bacteria	10112	0	0	-
Trichodesmium erythraeum IMS101	203124	Bacteria	10069	0	0	-
Serratia proteamaculans 568	399741	Bacteria	9844	0	1	0
Desulfitobacterium hafniense DCB-2	272564	Bacteria	9706	0	1	0
Sinorhizobium medicae WSM419	366394	Bacteria	9668	0	0	-
Vibrio vulnificus	672	Bacteria	9621	0	1	0
Pseudomonas putida GB-1	76869	Bacteria	9563	0	1	0
Burkholderia thailandensis E264	271848	Bacteria	9472	0	0	-
Bacillus subtilis	1423	Bacteria	9465	0	0	-
Vibrio sp. Ex25	150340	Bacteria	9385	0	1	0
Salmonella enterica subsp. enterica serovar Typhi	90370	Bacteria	9305	0	2	0
Pseudomonas putida F1	351746	Bacteria	9305	0	1	0
Streptomyces coelicolor	1902	Bacteria	9166	0	0	-
Pseudomonas syringae pv. phaseolicola 1448A	264730	Bacteria	8951	0	0	-
Vibrio splendidus LGP32	575788	Bacteria	8930	0	0	-
Pectobacterium carotovorum subsp. carotovorum PC1	561230	Bacteria	8921	0	1	0
Escherichia coli K-12	83333	Bacteria	8897	0	4	0
Escherichia coli IAI1	585034	Bacteria	8818	0	4	0
Vibrio cholerae	666	Bacteria	8730	0	0	-
Escherichia coli ATCC 8739	481805	Bacteria	8635	0	4	0
Chloroflexus aurantiacus J-10-fl	324602	Bacteria	8475	0	0	-
Salmonella enterica subsp. enterica serovar Paratyphi A str. AKU_12601	554290	Bacteria	8430	0	1	0
Shewanella putrefaciens CN-32	319224	Bacteria	8414	0	0	-
Escherichia coli str. K-12 substr. DH10B	316385	Bacteria	8185	0	4	0
Proteus mirabilis HI4320	529507	Bacteria	8182	0	1	0
Pseudomonas mendocina ymp	399739	Bacteria	8102	0	0	-
Salmonella enterica subsp. enterica serovar Paratyphi A	54388	Bacteria	8067	0	1	0

Bordetella bronchiseptica	518	Bacteria	7995	0	0	-
Robiginitalea biformata HTCC2501	313596	Bacteria	7867	0	0	-
Vibrio cholerae O395	345073	Bacteria	7785	0	0	-
Acinetobacter sp. ADP1	62977	Bacteria	7717	0	0	-
Polaromonas naphthalenivorans CJ2	365044	Bacteria	7712	0	0	-
Alteromonas macleodii str. 'Deep ecotype'	314275	Bacteria	7656	0	0	-
Azotobacter vinelandii DJ	322710	Bacteria	7593	0	0	-
Streptococcus pneumoniae	1313	Bacteria	7559	0	0	-
Burkholderia mallei NCTC 10229	412022	Bacteria	7470	0	0	-
Burkholderia mallei	13373	Bacteria	7453	0	0	-
Synechocystis sp. PCC 6803	1148	Bacteria	7362	0	0	-
Mycobacterium gilvum PYR-GCK	350054	Bacteria	7362	0	1	0
Clostridium botulinum B str. Eklund 17B	508765	Bacteria	7335	0	0	-
Rhodopseudomonas palustris BisB18	316056	Bacteria	7299	0	0	-
Chelativorans sp. BNC1	266779	Bacteria	7155	0	0	-
Burkholderia mallei SAVP1	320388	Bacteria	7134	0	0	-
Clostridium thermocellum ATCC 27405	203119	Bacteria	7123	0	0	-
Paracoccus denitrificans PD1222	318586	Bacteria	7026	0	1	0
Xanthobacter autotrophicus Py2	78245	Bacteria	6967	0	0	-
Treponema denticola	158	Bacteria	6964	0	0	-
Clostridium botulinum E3 str. Alaska E43	508767	Bacteria	6888	0	0	-
Clostridium perfringens	1502	Bacteria	6623	0	0	-
Aromatoleum aromaticum EbN1	76114	Bacteria	6569	0	0	-
Methylibium petroleiphilum PM1	420662	Bacteria	6395	0	0	-
Geobacillus sp. WCH70	471223	Bacteria	6333	0	0	-
Listeria innocua	1642	Bacteria	6008	0	0	-
Clostridium tetani	1513	Bacteria	5885	0	0	-
Listeria monocytogenes HCC23	552536	Bacteria	5832	0	0	-
Listeria welshimeri serovar 6b str. SLCC5334	386043	Bacteria	5670	0	0	-
Bordetella avium 197N	360910	Bacteria	5575	0	0	-
Bordetella pertussis	520	Bacteria	5323	0	0	-
Helicobacter pylori	210	Bacteria	5285	0	0	-
Hyphomonas neptunium ATCC 15444	228405	Bacteria	5112	0	0	-
Corynebacterium glutamicum	1718	Bacteria	5071	0	1	0
Pasteurella multocida	747	Bacteria	5033	0	0	-
Streptococcus mutans	1309	Bacteria	4913	0	0	-
Brucella melitensis	29459	Bacteria	4906	0	1	0
Porphyromonas gingivalis	837	Bacteria	4868	0	0	-
Brucella canis ATCC 23365	483179	Bacteria	4859	0	2	0
Mannheimia succiniciproducens MBEL55E	221988	Bacteria	4837	0	0	-

Brucella suis	29461	Bacteria	4827	0	2	0
Clostridium novyi NT	386415	Bacteria	4761	0	0	-
Brucella suis ATCC 23445	470137	Bacteria	4747	0	2	0
Brucella abortus S19	430066	Bacteria	4557	0	1	0
Brucella abortus	235	Bacteria	4518	0	1	0
Brucella melitensis biovar Abortus 2308	359391	Bacteria	4428	0	2	0
Corynebacterium glutamicum R	340322	Bacteria	4426	0	1	0
Brucella ovis ATCC 25840	444178	Bacteria	4296	0	2	0
Moorella thermoacetica ATCC 39073	264732	Bacteria	4292	0	0	-
Corynebacterium efficiens	152794	Bacteria	4249	0	0	-
Campylobacter lari RM2100	306263	Bacteria	4188	0	0	-
Streptococcus pneumoniae D39	373153	Bacteria	4162	0	0	-
Haemophilus somnus 129PT	205914	Bacteria	4027	0	0	-
Helicobacter pylori P12	570508	Bacteria	3971	0	0	-
Streptococcus pyogenes serotype M6	301450	Bacteria	3874	0	0	-
Borrelia burgdorferi	139	Bacteria	3768	0	0	-
Streptococcus pyogenes str. Manfredo	160491	Bacteria	3634	0	0	-
Mycoplasma penetrans	28227	Bacteria	3412	0	0	-
Rickettsia bellii RML369-C	336407	Bacteria	3412	0	0	-
Rickettsia felis	42862	Bacteria	3406	0	0	-
Corynebacterium diphtheriae	1717	Bacteria	3389	0	1	0
Haemophilus ducreyi	730	Bacteria	3366	0	0	-
Neisseria meningitidis serogroup A	65699	Bacteria	3362	0	0	-
Neisseria meningitidis serogroup B	491	Bacteria	3343	0	0	-
Borrelia garinii	29519	Bacteria	3269	0	0	-
Borrelia burgdorferi ZS7	445985	Bacteria	3215	0	0	-
Neisseria gonorrhoeae FA 1090	242231	Bacteria	3190	0	0	-
Rickettsia bellii OSU 85-389	391896	Bacteria	3174	0	0	-
Methanothermobacter thermautotrophicus str. Delta H	187420	Bacteria	3016	0	0	-
Acidothermus cellulolyticus 11B	351607	Bacteria	2971	0	0	-
Chlamydia pneumoniae	83558	Bacteria	2805	0	0	-
Orientia tsutsugamushi str. Ikeda	334380	Bacteria	2796	0	0	-
Dehalococcoides ethenogenes 195	243164	Bacteria	2509	0	0	-
Orientia tsutsugamushi str. Boryong	357244	Bacteria	2493	0	0	-
Chlamydophila abortus	83555	Bacteria	2472	0	0	-
Rickettsia akari str. Hartford	293614	Bacteria	2458	0	0	-
Chlamydia trachomatis	813	Bacteria	2273	0	0	-
Treponema pallidum	160	Bacteria	2224	0	0	-
Chlamydia trachomatis A/HAR-13	315277	Bacteria	2213	0	0	-
Mycoplasma agalactiae PG2	347257	Bacteria	2073	0	0	-

Anaplasma phagocytophilum HZ	212042	Bacteria	2009	0	0	-
Anaplasma marginale str. Florida	320483	Bacteria	1958	0	0	-
Mycoplasma pneumoniae	2104	Bacteria	1884	0	0	-
Mycoplasma genitalium	2097	Bacteria	1590	0	0	-
Human herpesvirus 6 (strain Uganda-1102)	10370	Viruses	400	0	0	-
Human herpesvirus 7 strain JI	57278	Viruses	359	0	0	-
Human herpesvirus 2 strain HG52	10315	Viruses	202	0	0	-
Epstein-barr virus strain ag876	82830	Viruses	153	0	1	0
Porcine circovirus 2	85708	Viruses	149	0	0	-
Human coronavirus OC43	31631	Viruses	140	0	0	-
Porcine transmissible gastroenteritis coronavirus strain Purdue	11151	Viruses	113	0	0	-
Murine hepatitis virus strain JHM	11144	Viruses	109	0	0	-
Feline infectious peritonitis virus (strain 79-1146)	33734	Viruses	108	0	0	-
Murine hepatitis virus strain A59	11142	Viruses	105	0	0	-
Escherichia coli ETEC H10407	316401	Bacteria	100	0	0	-
Human coronavirus 229E	11137	Viruses	95	0	0	-
Human adenovirus 2	10515	Viruses	93	0	0	-
Avian infectious bronchitis virus (strain M41)	11127	Viruses	92	0	0	-
Porcine epidemic diarrhea virus (strain CV777)	229032	Viruses	92	0	0	-
West Nile virus	11082	Viruses	83	0	0	-
Simian rotavirus A/SA11-both	37137	Viruses	65	0	0	-
Human rotavirus strain P	10957	Viruses	64	0	0	-
Human rotavirus strain WA	10962	Viruses	63	0	0	-
Bovine rotavirus strain RF	10933	Viruses	60	0	0	-
Borna disease virus	12455	Viruses	57	0	0	-
Nipah virus	121791	Viruses	52	0	0	-
JC polyomavirus	10632	Viruses	48	0	0	-
Bluetongue virus (serotype 10 / American isolate)	10900	Viruses	45	0	0	-
Lelystad virus	11049	Viruses	45	0	0	-
Sonchus yellow net virus	11307	Viruses	40	0	0	-
Bovine herpesvirus type 1.1 (strain Cooper)	10323	Viruses	37	0	0	-
Human papillomavirus type 35	10587	Viruses	31	0	0	-
Human papillomavirus type 56	10596	Viruses	31	0	0	-
Rabies virus ERA	11295	Viruses	31	0	0	-
Whitewater Arroyo virus	46919	Viruses	31	0	0	-
Simian immunodeficiency virus - mac K6W	11735	Viruses	30	0	0	-
Human papillomavirus type 18	333761	Viruses	30	0	0	-
Puumala virus (strain sotkamo/v-2969/81)	39002	Viruses	29	0	0	-
Simian immunodeficiency virus (MM142-83 ISOLATE)	11733	Viruses	28	0	0	-

Oliveros virus	42764	Viruses	28	0	0	-
Human papillomavirus type 58	10598	Viruses	27	0	0	-
Rift valley fever virus (STRAIN ZH-548 M12)	11589	Viruses	27	0	0	-
Blackcurrant reversion virus	65743	Viruses	27	0	0	-
Human papillomavirus type 39	10588	Viruses	26	0	0	-
Human papillomavirus type 45	10593	Viruses	26	0	0	-
Human papillomavirus type 6b	10600	Viruses	26	0	0	-
Western equine encephalomyelitis virus	11039	Viruses	26	0	0	-
Human immunodeficiency virus type 2 (isolate KR)	73484	Viruses	25	0	0	-
Human papillomavirus type 53	333765	Viruses	25	0	0	-
Woodchuck hepatitis virus 8	10433	Viruses	23	0	0	-
Human papillomavirus type 31	10585	Viruses	23	0	0	-
Human papillomavirus type 33	10586	Viruses	23	0	0	-
Human papillomavirus type 51	10595	Viruses	23	0	0	-
Human immunodeficiency virus type 2 (ISOLATE D194)	11713	Viruses	23	0	0	-
Human immunodeficiency virus type 2 (ISOLATE SBLISY)	11718	Viruses	23	0	0	-
Human immunodeficiency virus type 2 (ISOLATE GHANA-1)	11717	Viruses	22	0	0	-
Plum pox virus (isolate NAT)	12213	Viruses	22	0	0	-
Bean-pod mottle virus (strain Kentucky G7)	31715	Viruses	22	0	0	-
Human immunodeficiency virus type 2 (ISOLATE CAM2)	11715	Viruses	21	0	0	-
Human papillomavirus type 1a	10583	Viruses	20	0	0	-
Semliki forest virus	11033	Viruses	20	0	0	-
Human immunodeficiency virus type 2 (ISOLATE BEN)	11714	Viruses	20	0	0	-
Human papillomavirus type 44	10592	Viruses	19	0	0	-
Human rhinovirus A2	12130	Viruses	19	0	0	-
Human papillomavirus type 29	37112	Viruses	19	0	0	-
Human papillomavirus type 52	10618	Viruses	18	0	0	-
Kunjin virus (STRAIN MRM61C)	11078	Viruses	18	0	0	-
Theiler's encephalomyelitis virus (STRAIN BEAN 8386)	12125	Viruses	18	0	0	-
Theiler's encephalomyelitis virus (STRAIN DA)	12126	Viruses	18	0	0	-
Venezuelan equine encephalitis virus (strain Trinidad donkey)	11038	Viruses	16	0	0	-
Dengue virus 2 Thailand/NGS-C/1944	11065	Viruses	16	0	0	-
Dengue virus 2 Jamaica/1409/1983	11064	Viruses	14	0	0	-
Mengo virus	12107	Viruses	14	0	0	-
Dengue virus 2 Thailand/16681/84	31634	Viruses	14	0	0	-
Dengue virus 2 16681-PDK53	31635	Viruses	14	0	0	-
Murray valley encephalitis virus (strain MVE-1-51)	301478	Viruses	14	0	0	-
Human papillomavirus type 7	10620	Viruses	13	0	0	-
AKR (endogenous) murine leukemia virus	11791	Viruses	13	0	0	-

Dengue virus 1 Singapore/S275/1990	33741	Viruses	13	0	0	-
Hamster polyomavirus	10626	Viruses	12	0	0	-
Dengue virus 2 Puerto Rico/PR159-S1/1969	11066	Viruses	12	0	0	-
Japanese encephalitis virus strain JAOARS982	11075	Viruses	11	0	0	-
Adeno-associated virus - 2	10804	Viruses	10	0	0	-
Friend murine leukemia virus (ISOLATE FB29)	11797	Viruses	9	0	0	-
Heron hepatitis B virus	28300	Viruses	9	0	0	-
