# Supplementary material for: Genome-wide structural modelling of TCR-pMHC interactions
Source: BMC Genomics. 2013 Oct 16;14(Suppl 5):S5. doi: 10.1186/1471-2164-14-S5-S5 (PMC3852114; doi:10.1186/1471-2164-14-S5-S5)
Supplement: Additional file 8 — Comparisons between iMatrix and one-matrix of three MHC class I alleles on 389 complete pathogen database. Three MHC class I alleles are HLA-A0201 (Homo sapiens), H-2-Kb (Mus musculus), and H-2-Ld (Mus musculus). [file 1471-2164-14-S5-S5-S8.pdf]

Table S6 - Comparisons between *i*Matrix and one-matrix of three MHC class I alleles on 389 complete pathogen database

MHC allele	*i*Matrix				One-matrix				(A)/(B)
	No. of hits **(A)**	Positive hits	Negative hits	Precision	No. of hits **(B)**	Positive hits	Negative hits	Precision	
HLA-A0201	701,897	360	187	0.66	511,587	265	135	0.66	1.37
H-2-Kb	933,281	44	91	0.33	537,425	28	69	0.29	1.74
H-2-Ld	386,429	119	63	0.65	264,302	97	49	0.66	1.46
